# Towards a Global Barcode Library for *Lymantria* (Lepidoptera: Lymantriinae) Tussock Moths of Biosecurity Concern

**DOI:** 10.1371/journal.pone.0014280

**Published:** 2010-12-09

**Authors:** Jeremy R. deWaard, Andrew Mitchell, Melody A. Keena, David Gopurenko, Laura M. Boykin, Karen F. Armstrong, Michael G. Pogue, Joao Lima, Robin Floyd, Robert H. Hanner, Leland M. Humble

**Affiliations:** 1 Forest Sciences, University of British Columbia, Vancouver, British Columbia, Canada; 2 Entomology, Royal British Columbia Museum, Victoria, British Columbia, Canada; 3 Entomology, Australian Museum, Sydney, New South Wales, Australia; 4 Northern Research Station, United States Department of Agriculture, Hamden, Connecticut, United States of America; 5 Wagga Wagga Agricultural Institute, Industry & Investment NSW, Wagga Wagga, New South Wales, Australia; 6 Bio-Protection Research Centre, Lincoln University, Christchurch, New Zealand; 7 Systematic Entomology Laboratory, United States Department of Agriculture, Washington, D.C., United States of America; 8 Integrative Biology, University of Guelph, Guelph, Ontario, Canada; 9 Canadian Forest Service, Natural Resources Canada, Victoria, British Columbia, Canada; Montreal Botanical Garden, Canada

## Abstract

**Background:**

Detecting and controlling the movements of invasive species, such as insect pests, relies upon rapid and accurate species identification in order to initiate containment procedures by the appropriate authorities. Many species in the tussock moth genus *Lymantria* are significant forestry pests, including the gypsy moth *Lymantria dispar* L., and consequently have been a focus for the development of molecular diagnostic tools to assist in identifying species and source populations. In this study we expand the taxonomic and geographic coverage of the DNA barcode reference library, and further test the utility of this diagnostic method, both for species/subspecies assignment and for determination of geographic provenance of populations.

**Methodology/Principal Findings:**

Cytochrome oxidase I (COI) barcodes were obtained from 518 individuals and 36 species of *Lymantria*, including sequences assembled and generated from previous studies, vouchered material in public collections, and intercepted specimens obtained from surveillance programs in Canada. A maximum likelihood tree was constructed, revealing high bootstrap support for 90% of species clusters. Bayesian species assignment was also tested, and resulted in correct assignment to species and subspecies in all instances. The performance of barcoding was also compared against the commonly employed NB restriction digest system (also based on COI); while the latter is informative for discriminating gypsy moth subspecies, COI barcode sequences provide greater resolution and generality by encompassing a greater number of haplotypes across all *Lymantria* species, none shared between species.

**Conclusions/Significance:**

This study demonstrates the efficacy of DNA barcodes for diagnosing species of *Lymantria* and reinforces the view that the approach is an under-utilized resource with substantial potential for biosecurity and surveillance. Biomonitoring agencies currently employing the NB restriction digest system would gather more information by transitioning to the use of DNA barcoding, a change which could be made relatively seamlessly as the same gene region underlies both protocols.

## Introduction

Non-indigenous insects and the pathogens they harbour have an overwhelming ecological, socio-economic, and evolutionary impact on the forest ecosystems they invade [1]–[6]. The rapid initiation of containment and eradication programs is imperative to prevent the establishment and spread of adventive populations or individuals, which in turn relies on early detection and accurate identification of non-indigenous species as they enter a new region [7]. Surveillance and monitoring at or near sites considered high risk (e.g. ports, airports, cargo facilities) serve this function, but current practices have proven insufficient, as evidenced by the alarming increase in non-indigenous insect establishments in recent decades [8]. Species identification in particular is a vulnerable step, as suggested by studies documenting the identification rate of port interceptions (e.g. 40–100% incomplete identifications across families of Coleoptera [9]).

To aid in the inherently difficult task of species identification for biosecurity, several molecular approaches are described in the recent standard released by the International Plant Protection Convention on the diagnostic protocols for regulated pests [10]. In contrast to the many ad-hoc protocols recognized in this standard (e.g. allozymes, restriction/amplified fragment length polymorphism analysis, microsatellites—techniques are reviewed in [11]), DNA barcoding provides a standardized and generic platform while still meeting and exceeding the rigorous standards of data quality and transparency (reviewed by [12]). Several studies demonstrate the efficacy of the approach as applied to invasive species detection and determination of native provenance, including work on leeches [13], agromyzid leafminers [14], tephritid fruit flies [15], [16], ants [17], siricid wasps [18], true bugs [19], the cactus moth [20], the European poplar shoot borer [21], and nocturnal moths [22]. The integration of DNA barcoding into national biosurveillance programs has been protracted, but acceptance by certain agencies is apparent (e.g. see LBAM ID, which incorporates barcoding into the diagnostics of light brown apple moth and related species in California [23].

**Figure 1:**
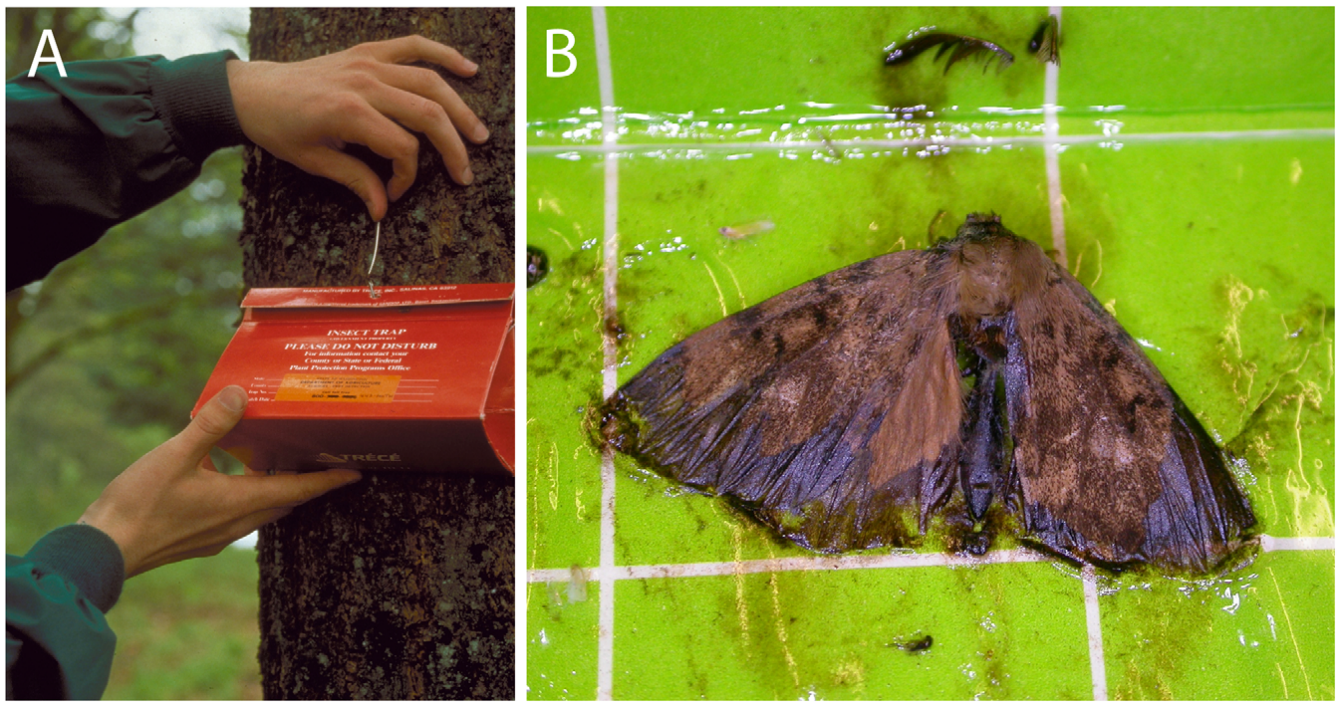
Collection of specimens through a pheromone-based gypsy moth surveillance program. A, Pheromone trap and B, trapped and damaged *Lymantria dispar* specimen (LMHRG001-06) (image A from USDA APHIS PPQ Archive, USDA APHIS PPQ, Bugwood.org).

The tussock moth genus *Lymantria* L. (Lepidoptera: Noctuoidea: Lymantriinae) has been the focus of numerous investigations of molecular diagnostic tools, predominantly on *Lymantria dispar* L. to separate the North American, European and Asian gypsy moth populations (e.g. [24]–[32]). The intensity of research effort is certainly warranted – few groups of insects rival the tussock moths in number of undesirable, potentially invasive species and the destruction they wreak on both native and non-native forests. This invasive potential is the product of several undesirable life history traits including long overwintering period in the egg stage, polyphagy in the larval stage, attraction of adults to artificial light sources (e.g. of ports and cargo vessels), and oviposition by females on inanimate surfaces [32]–[34]. Invasive species identification for this group is complicated by the propensity for immatures to be the stage of translocation, providing few characters for morphological diagnosis. Furthermore, when the adults are targeted, as is the case with traps employing synthetic sex pheromone attractants, lures may inadvertently attract more then one species (e.g. [35]), and individuals may be severely damaged by the trapping mechanism (e.g. sticky traps; see Figure 1).

Two previous studies [15], [36] initiated a DNA barcode library for *Lymantria* (and other tussock moth genera) and results suggested potential for biosurveillance and species identification. The present study builds upon this solid foundation, to further populate the library with additional species, test that its utility remains with comprehensive sampling within species, and employ the reference library to assign intercepted specimens from quarantine and monitoring programs in British Columbia, Canada. In addition, we compare the DNA barcoding approach to a diagnostic assay based on the same gene region, herein called the ‘NB system’, routinely-used in surveillance programs to assess the gross geographic provenance of *Lymantria dispar* [24], [26], and suggest a simple transition for monitoring programs to adopt the barcoding approach with its improved reliability and resolution.

## Materials and Methods

An effort was made to expand the taxonomic and geographic coverage of the DNA barcode library for the tussock moth genus *Lymantria*, with particular attention to those at high risk of being transported in cargo or on vessels to new habitats where they could become invasive pests, recently reviewed by Pogue and Schaefer [34]. We followed the taxonomy of [34] and [37], with the exceptions of *L. nebulosa* Wileman and *L. subpallida* Okano, which are considered here as species (see Appendix S1 for taxonomic treatment), and identifications were determined using these monographs. Samples and sequences were obtained from four sources. Firstly, sequences were obtained from several previous studies that analyzed all or part of the barcode region: Armstrong & Ball [15] (N = 76), Ball & Armstrong [36] (5), Bogdanowicz et al. [26] (74), Hebert et al. [38] (12), and Yamaguchi et al. unpublished (8). Secondly, 146 samples were analysed from vouchered material in publicly accessible collections, namely the Agricultural Scientific Collections Trust (ASCT, New South Wales, Australia), Biodiversity Institute of Ontario (Guelph, Canada), Pacific Forestry Centre (Victoria, Canada), Smithsonian National Museum of Natural History (Washington D.C., United States), and University of Maryland Alcohol Tubes of Lepidoptera collection (College Park). Thirdly, 165 specimens were analyzed from the USDA Forest Service Northern Research Station (Hamden, United States) which included representatives from all 46 populations of *Lymantria dispar* analyzed in Keena et al. [32]. Finally, 32 specimens were acquired from surveillance programs in British Columbia, Canada. Thirty of these specimens were collected in sticky traps baited with *L. dispar* pheromone (Figure 1) around the province during 2006 and 2009 and two individuals were reared from egg masses removed from a Russian cargo vessel in the port of Vancouver in 1992.

**Figure 2:**
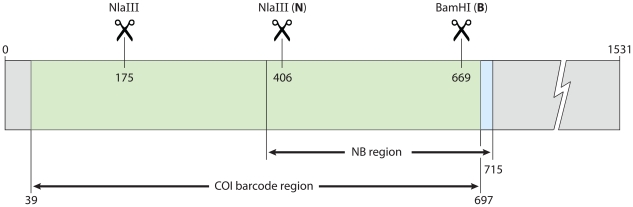
Barcode region of the cytochrome oxidase I (COI) gene. The barcode region spans 658 base pairs near to the 5′ end of the COI gene and includes the two diagnostic restriction enzyme sites of the ‘NB system’ of Bogdanowicz et al. [24], referred to as N and B. An additional monomorphic NlaIII site is found in the 5′ region of the gene fragment and therefore a NlaIII enzyme digest of the barcode region would result in two (N−) or three (N+) bands (instead of 1 or 2 bands).

DNA extraction, amplification, and sequencing of the barcode region of the mitochondrial cytochrome oxidase I (COI) gene followed standard high-throughput DNA barcoding methods previously described [38], [40]. The primers LepF1 and LepR1 [41] were used in most instances, but amplification and sequencing using the ‘Lep mini primers’ (MLepF1, MLepR1) [42] was necessary for a few older specimens. Molecular work performed in Australia used the COI primers and amplification methods described in Cho et al. [43]. All sequences are publicly available from the Barcode of Life Data Systems (BOLD) (www.boldsystems.org; [44]; project ‘Global Lymantria’ [GLYMA]) and GenBank (see Table S1 for accession numbers). The 658 base pair (bp) barcode region encompasses 360 of the 378 bp amplicon and the two restriction enzyme sites of the ‘NB system’ of Bogdanowicz et al. [24] that is routinely used for gypsy moth diagnostics (Figure 2). The four possible haplotypes in this system are N+B+, N−B+, N+B−, and N−B−, where presence (+) or absence (−) is indicated for the NlaIII and BamHI restriction sites (Table 1).

**Table 1:** Key to the ‘NB system’ routinely used for gypsy moth diagnostics.

Fragment size after NlaIII (N) enzyme digest	Fragment size after BamHI (B) enzyme digest	NB haplotype
∼350 bp	∼400 bp	N+ B−
∼350 bp	∼360 bp	N+ B+
∼400 bp	∼400 bp	N− B−
∼400 bp	∼360 bp	N− B+

The two diagnostic restriction enzyme digests of Bogdanowicz et al. [24] can result in the four haplotypes listed.

To explore the utility of the barcode region for species diagnosis in *Lymantria*, a maximum likelihood tree was constructed in Garli 1.0 [45], using the best-fit model as determined by ModelTest 3.7 [46] under the Akaike Information Criterion. The general-time-reversible substitution model was chosen, with among-site-rate-heterogeneity modeled according to a gamma distribution, and an estimated proportion of DNA sites invariant. Analysis used default settings with *Orgyia antiqua* (BOLD ProcessID XAG647-05, GenBank Accession GU091296) included as an outgroup; node supports were estimated using 100 bootstrap replicates. The levels of genetic variation within and between species were calculated in MEGA 4 [47] using pairwise Kimura 2-parameter (K2P) distances [48] and the pairwise deletion option. The number of morphologically defined species that could successfully be differentiated by DNA barcoding were tallied to determine an overall success rate. Successful differentiation of a species required that its barcodes formed monophyletic clusters and were not shared with other species; in the case of species with a single representative, only the latter criterion was necessary.

To investigate subspecies delimitation in *Lymantria dispar* using the COI gene, we parsed the dataset to the 244 sequences >600 bp. Removing the significant sequence length variation permitted the use the more rapid neighbour-joining (NJ) method. The NJ tree was constructed in MEGA 4 with K2P distances and the pairwise deletion option (and resulted in a near-identical topology to an ML analysis; data not shown). Specimens were classified to subspecies based on the distribution limits provided by Pogue and Schaefer [34] where sufficient locality data were available.

For both the species and subspecies level, Bayesian assignment tests were performed using the ‘segregating sites’ algorithm of Abdo and Golding [49] (see also [48]). For all species with three or more individuals, one randomly-chosen COI sequence was removed from the dataset and used as the query sequence for the test, as in Taveres and Baker [51]. This procedure was subsequently repeated for all haplotypes of the 32 surveillance specimens. The number of segregating sites, the posterior probability of correct assignment, and the risk of mis-assignment were calculated with the program Assigner (available at http://info.mcmaster.ca/TheAssigner/; [49]). The assignment of species and subspecies (and potential for determining geographic provenance and female flight ability) with the COI barcode region was compared against the commonly employed ‘NB system’ [24].

## Results

COI barcodes were obtained from 518 individuals and 36 species of *Lymantria*, originating from 35 countries (Table S1). Sixteen species were represented by a single sample sequence while the remaining species ranged from 2 to 308 sequences (mean excluding *L. dispar*  = 5.8 sequences/species). The average sequence length was 597 bp despite several shorter sequences obtained from previous studies (e.g. sequences from [26] are 375–378 bp). Four species (*L. singapura*, *L. semperi*, *L. todara*, and *L. subrosea*) had sequences shorter than half the barcode region due to specimen age and/or preservation. There was no evidence of insertions, deletions or stop codons detected, suggesting pseudogenes were absent from the dataset.

**Figure 3:**
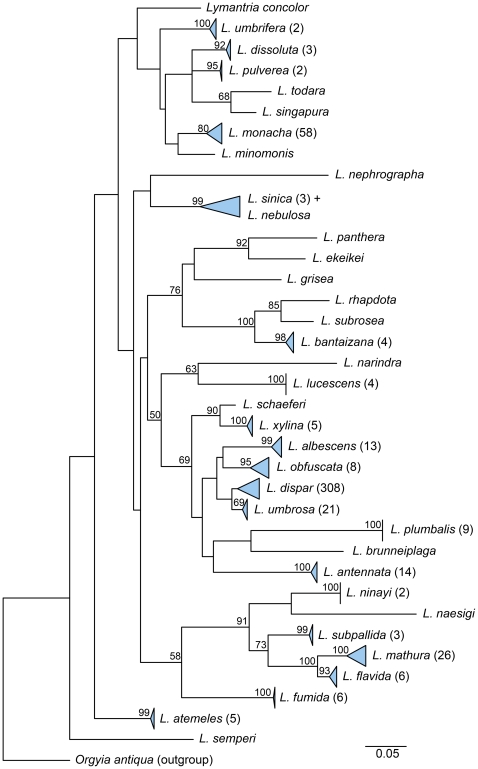
Maximum likelihood tree for 36 species of *Lymantria*. Tree was constructed with the barcode region of the COI gene for 518 individuals. The number of specimens collapsed into a single node is given in parentheses after the taxon name. Bootstrap support values >50% are listed above the corresponding node. Width of the triangles represents the sequence divergence within the cluster. Refer to Figure S1 for full tree.

**Figure 4:**
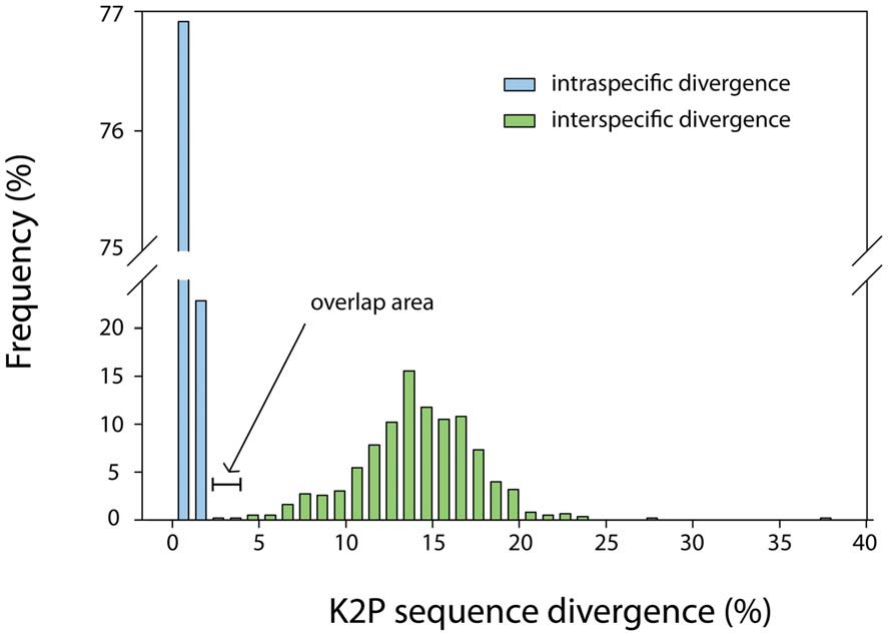
Combined histograms of pairwise Kimura 2-Parameter (K2P) sequence variation. Blue vertical bars show intraspecific divergences for the 20 species of *Lymantria* with multiple individuals and green vertical bars show the interspecific divergences between all 36 species.

The ML tree for the 36 species revealed long branches between the terminal clusters of closely related haplotypes (Figure 3, Figure S1). In only one case did a terminal cluster not correspond to known species limits: *L. sinica* (n = 3) was paraphyletic with respect to the single specimen of *L. nebulosa*, with the deep split in *L. sinica* equalling 2.9% K2P sequence divergence (LYMAN070-08, locality: China split from LYMAN065-08 & LYMAN066-08, locality: Taiwan). Eighteen of the 20 species clusters with multiple individuals had high bootstrap support (>80%); the remaining two species, *L. umbrosa* and *L. dispar*, considered conspecific until recently [34] and respond to the same pheromone lure [35], had bootstrap support of 69% and <50%, respectively. The mean K2P sequence divergence between species (x = 14.02%; SD  = 3.47%; range  = 2.68–37.61%) was approximately 21-fold higher than within species variation (0.66%; SD  = 0.50%; range  = 0–3.38%) and formed mostly disjunct distributions (Figure 4). Only 157 of 50,390 intraspecific comparisons and 2 of 630 interspecific comparisons fell within a small range of overlap, between 2 and 4%. The former cases involved *L. mathura*, *L. sinica*, *L. obfuscata*, and *L. dispar* (only those comparisons involving one or two short GenBank sequences) and the latter involved the pairs *L. dispar/umbrosa* and *L. xylina/schaeferi*. Overall, DNA barcoding was able to successfully differentiate 35 of 36 (97.2%) morphologically defined species.

**Figure 5:**
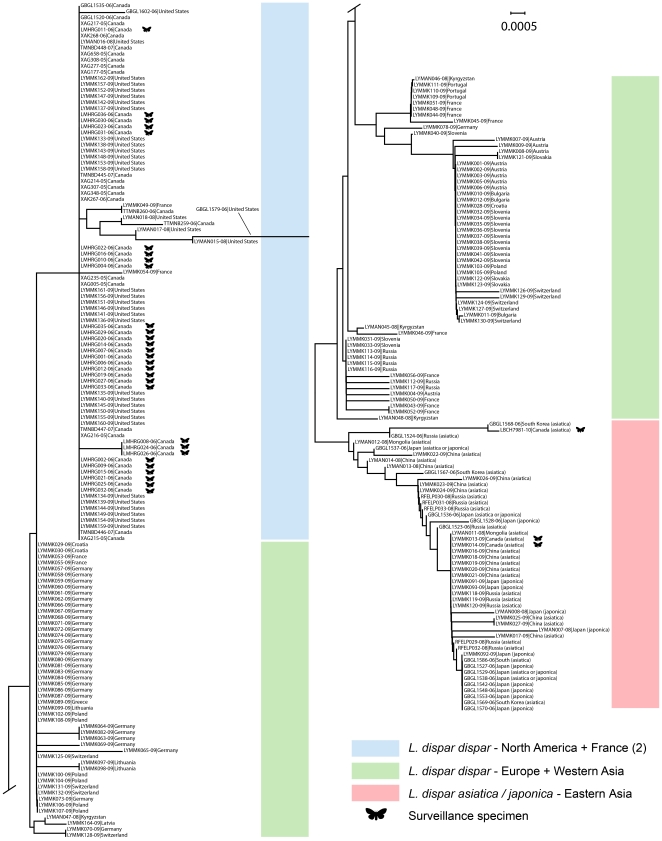
Neighbour-joining tree of the gypsy moth *Lymantria dispar*. Tree was constructed with 244 sequences of the COI barcode region. BOLD process IDs and collection localities are provided for each sequence. Surveillance specimens are denoted by a moth symbol.

The NJ tree of COI sequence divergences in *L. dispar* indicated a clear delineation between the *L. d. dispar* subspecies and the two Asian subspecies *asiatica* and *japonica* (Figure 5). The tree also revealed all North American *L. d. dispar* individuals clustered together along with two sequences from France (LYMMK049-09, LYMMK054-09). The 32 specimens caught in surveillance programs in British Columbia, Canada comprised four COI haplotypes. Two of these haplotypes clustered within the North American *L. d. dispar*, while the other two clustered in the *asiatica/japonica* group—in all 32 cases, there is perfect correspondence with the subspecies designations deduced from morphology and the origin/pathway of introduction.

The tests of assignment in a Bayesian statistical framework resulted in correct assignment to species and subspecies in all instances (Table 2). The posterior probabilities of assignment for most species were high, and the risks of mis-assignment were low. The assignment of surveillance specimens to *L. dispar* and their respective subspecies displayed substantially lower posterior probabilities and higher risks, likely a product of the close association of *L. dispar* with *L. umbrosa*, as well as the nonmonophyly of *L. d. asiatica* and *japonica.*

**Table 2:** Assignment of random and surveillance individuals to species and sub-species.

Query	Query taxon	Process ID	No. of individuals	No. of diagnostic sites	Posterior probability	Risk
**species**	*L. mathura*	LYMAN019	26	14	0.998	3.29×10−5
	*L. monacha*	LYMMK174	58	13	0.974	2.75×10−4
	*L. antennata*	LYMAN174	3	5	0.987	6.09×10−5
	*L. sinica*	LYMAN066	3	11	0.997	8.73×10−5
	*L. obfuscata*	LYMAN074	8	13	0.897	2.86×10−3
	*L. dissoluta*	LYMAN055	3	2	0.960	1.21×10−4
	*L.* subpallida	LYMAN025	3	2	0.992	3.87×10−5
	*L. bantaizana*	GBGL1580	4	2	0.986	1.09×10−4
	*L. lucescens*	GBGL1613	4	0	0.994	0
	*L. atemeles*	LYMAN042	5	3	0.984	4.73×10−5
	*L. xylina*	GBGL1587	5	5	0.85	9.14×10−4
	*L. flavida*	LYMAN028	6	6	0.983	1.25×10−4
	*L. fumida*	GBGL1589	6	1	0.993	9.92×10−6
	*L. plumbalis*	LYMAN108	9	0	0.998	0
	*L. albescens*	LYMAN033	13	5	0.697	1.85×10−3
	*L. umbrosa*	LYMMK095	21	12	0.572	3.26×10−3
**surveillance - species**	*L. dispar*	LMHRG001	308	49	0.727	3.35×10−3
	*L. dispar*	LMHRG008	308	49	0.766	3.23×10−3
	*L. dispar*	LBCH7981	308	49	0.694	5.17×10−3
	*L. dispar*	LYMMK013	308	49	0.694	5.17×10−3
**surveillance - subspecies**	*L. d. dispar*	LMHRG001	203	29	0.593	3.10×10−3
	*L. d. dispar*	LMHRG008	203	29	0.656	3.15×10−3
	*L. d. asiatica*	LBCH7981	54	6	0.462	3.28×10−3
	*L. d. asiatica*	LYMMK013	54	2	0.302	1.06×10−3

Provided are the three query types, query taxon and Process ID assigned, number of individuals in query taxon, diagnostic sites, posterior probability of correct assignment, and risk of mis-assignment.

The haplotypes of the ‘NB system’ were inferred based on the COI sequence present at the two restriction sites (Table S1) rather than by restriction endonuclease digests. As in Keena et al. [32], the three haplotypes N+B+, N−B− and N+B− were present in *L. dispar*. All 3 haplotypes were also found in at least one other species—one species possessed N+B+, five species with N−B−, and 16 species scored as N+B−. Currently it is unclear exactly which species will generate a COI amplicon with the primers of Bogdanowicz et al. [24], [27], but previous work has successfully assayed *L. albescens* (*L. dispar albescens* in [26]), *L. umbrosa* (*L. dispar hokkaidoensis* in [27]), and *L. monacha* (LMH, unpublished data of male from Russian Far East trapped in greater Vancouver, Canada in 1992). This suggests that misidentification of species prior to the ‘NB system’ assay could have serious ramifications. For example, the heavily regulated nun moth (*L. monacha*), if captured in North America and inadvertently assayed as *L. dispar*, would be determined to possess the N+B− haplotype (Table S1), and mistakenly diagnosed as European gypsy moth, *L. dispar dispar*. In respect to tracing geographic origins, the 3 NB haplotype states remain mostly informative as originally characterized [24], but with limited resolution and outliers that could also be misleading (e.g. BOGDA004-08, from Ontario, Canada has the N+B− haplotype characteristic of Europe).

In contrast, the COI sequence data consisted of 91 haplotypes within *L. dispar* and 142 haplotypes across all species, none of which were shared between species. Moreover, the haplotype data recovered clusters of *L. d. dispar*, North American *L. d. dispar*, and *L. d. asiatica/japonica*. The presence of multiple barcode-determined haplotypes in several regions (e.g. 6 haplotypes from 7 individuals in the Indre-et-Loire department of France; 5 haplotypes from 9 individuals from the Ticino Canton in Switzerland) suggests the potential for determining the native provenance (and associated traits such as female flight ability) by analyzing haplotype frequencies across the native and introduced range (e.g. [20], [52], [19]). Such analyses may be compromised if human-associated movement has altered haplotype frequencies in the native populations, but this potential exists for all methods tracing source populations. Although the sampling density in the present study was not adequate for these analyses, the presence of two haplotypes from France which clustered within the North American *L. d. dispar* is consistent with the well-documented introduction of the species in 19^th^ century Massachusetts by Leopold Trouvelot [53].

## Discussion

This study has clearly demonstrated the efficacy of DNA barcodes for diagnosing species of *Lymantria* and has reinforced the view that the approach has substantial potential for biosecurity and surveillance [12]. The minimal overlap between mean intra- and interspecific variation resulted in well-supported, cohesive clusters of sequences corresponding with named species. In only one case—the paraphyly of *L. sinica* relative to *L. nebulosa*—did the COI barcodes fail to correctly distinguish a species, providing a success rate of 97.2% among 36 morphologically defined *Lymantria* species. This high success rate is comparable to three recent barcode studies of Lepidoptera: 100% success observed in a temperate, local study of 190 species [22]; 97.9% in a tropical, regional study of 521 species [42]; and 99.3% in a temperate, continental study of 1,327 species [38].

It remains possible that the deep split in *L. sinica* is genuine and reflects the allopatric divergence between the nominal species and a misidentified or undescribed species, or that *L. sinica* has maintained a deep COI polymorphism following its divergence from *L. nebulosa*. In any case, further investigation is warranted. Interestingly, a deep COI divergence revealed within *L. mathura* in the study of Ball and Armstrong [36] pointed to a possible cryptic species *L. flavida* by Pogue and Schaefer [34], explaining previously mixed responses to a synthetic pheromone lure. Likewise, our preliminary COI data identified a deep divergence within *L. mathura*, which led us to revise the status of *L. subpallida* (previously synonymized by [37]; see Appendix S1).

In addition to these instances of deep divergence, there are two pairs of species which show <2% sequence divergence. *L. dispar* and *L. umbrosa* were recognized as separate species by Pogue and Schaefer [34], contra Schintlmeister [37], based in large part on the DNA barcoding study of Ball and Armstrong [36], and their distributions, with *L. umbrosa* confined to the northern part of Hokkaido, Japan. *L. xylina* and *L. schaeferi* also show <2% sequence divergence, and again recent taxonomic studies disagree on their species status: [34] treated *L. xylina* from mainland China, Taiwan and Japan as conspecific but [37] described the Chinese populations as a new species, *L. schaeferi*. Our data confirms that these taxa are closely related, but given that we have sampled just a single specimen of the latter species, more data are needed to resolve this question. All these cases illustrate that DNA barcoding provides secondary benefits to biosecurity applications—taxonomic insights and implications for detection and monitoring in the field. It also validates a fundamental practice in barcoding projects, the retention of voucher specimens [12], to facilitate future taxonomic examination and to avoid the proliferation of incorrect identifications or ‘error cascades’ [54].

The positive results of the species and sub-species assignment tests, calculated for both reference and surveillance specimens, demonstrates the future potential for the application of DNA barcoding to biosecurity. An actively curated and validated reference library is queried with the DNA barcode obtained from a surveillance or quarantine specimen; within a robust statistical framework, species assignments are calculated along with measures of confidence; where sufficient coverage exists, additional assignments are computed (e.g. subspecies identification, native provenance, characteristics related to invasiveness), which can be corroborated with additional markers; voucher specimens are retained to facilitate morphological confirmation and future taxonomic enquiry. While the scaffold is in place for this at the present time (i.e. BOLD [44]), further benchmarking of statistical assignment programs is necessary (e.g. [50]), as is the continued construction and maintenance of reference barcode libraries. For the latter, participation by multiple national biosecurity research and monitoring programs is imperative.

**Figure 6:**
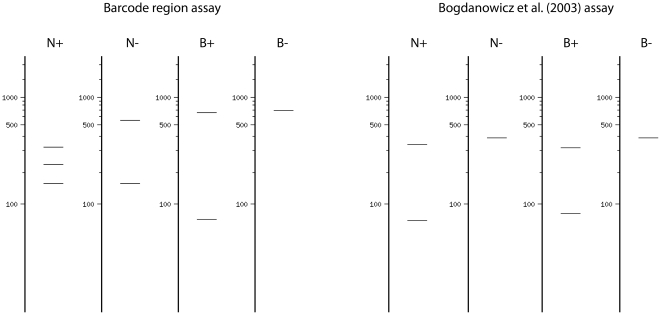
Simulated gel electrophoresis for two restriction fragment length polymorphism assays. The barcode region assay amplifies a ∼690 bp fragment, whereas the Bogdanowicz et al [24] assay amplifies ∼380 bp. The presence (+) or absence (−) of the NlaIII and BamHI restriction sites are provided for each assay, as described in the text. The digest and electrophoresis simulations were performed with New England BioLabs NEBcutter V2.0 (http://tools.neb.com/NEBcutter2).

To this end, our comparison of the commonly used ‘NB system’ [24] with the COI barcode approach clearly indicates that higher resolution and generality would be achieved by *Lymantria* monitoring programs should they adopt DNA barcoding. We have demonstrated instances when the ‘NB system’ would provide misleading results that could have serious consequences, whereas COI barcodes provide reliable subspecies and broad geographic information. Moreover, recent studies (e.g. [20], [52], [19]) indicate that higher sampling density should allow more precise tracing of source populations, which in turn will predict female flight ability, the key trait of interest [32]. Because the same COI gene region underlies both approaches, the transition for monitoring agencies could be seamless. Instead of amplifying the 378 bp fragment of Bogdanowicz et al. [24], the 658 bp barcode region could be amplified with the near-universal LepF1/LepR1 primers [41]. Assays of the NlaIII and BamHI enzyme digests could continue to be performed, producing slightly different banding patterns (Figure 6), but providing a rapid initial assessment as with the ‘NB system’. The amplicon could then be sequenced for definitive species and broad geographic information. The increased sampling density would continually develop a baseline from which progressively finer levels of information could be gleaned, then confirmed by other marker systems (e.g. FS1 [28] or microsatellites [24]) when necessary.

### Conclusion

In this study we have shown that DNA barcoding is an effective tool for discriminating species of *Lymantria*. Furthermore, our comparison of this system against the NB restriction digest system—commonly employed at present by biomonitoring agencies for this purpose—suggests that the informational value provided by barcoding (species resolution, general applicability) is significantly greater. DNA barcoding also has the potential to provide novel taxonomic insights, facilitated by its rigorous standards of record-keeping and permanent archiving of voucher specimens. As the DNA barcode reference library continues to increase in coverage, both for *Lymantria* and other taxa of international regulatory concern, its utility as part of a diagnostic/monitoring system will continue to expand.

## Supporting Information

Appendix S1 Taxonomic treatment of *Lymantria nebulosa* Wileman, revised status and *Lymantria subpallida* Okano, revised status.
(9.23 MB PDF)

Figure S1 Complete maximum likelihood tree for 36 species of *Lymantria*. Full tree from Figure 3 displaying haplotype variation within species clusters. BOLD process IDs and collection localities are provided for each sequence. Tree was rooted with the outgroup *Orgyia antiqua*.
(0.02 MB PDF)

Table S1 Taxonomic samples used in study, including Barcode of Life Database (BOLD) and GenBank accessions. Also listed is the presence (+) or absence (-) of the NlaIII (N) and BamHI (B) restriction enzyme sites of the ‘NB system’ [24], determined from the sequence data (i.e. not by performing the assay).
(0.16 MB PDF)

## References

[1] Liebhold AM, Macdonald WL, Bergdahl D, Mastro VC (1995). Invasion by exotic forest pests: A threat to forest ecosystems.. Forest Science Monographs.

[2] Mooney HA, Cleland EE (2001). The evolutionary impact of invasive species.. Proceedings of the National Academy of Sciences of the United States of America.

[3] Gurevitch J, Padilla DK (2004). Are invasive species a major cause of extinctions?. Trends in Ecology and Evolution.

[4] Pimentel D, Zuniga R, Morrison D (2005). Update on the environmental and economic costs associated with alien-invasive species in the United States.. Ecological Economics.

[5] Lovett GM, Canham CD, Arthur MA, Weathers KC, Fitzhugh RD (2006). Forest ecosystem responses to exotic pests and pathogens in eastern North America.. BioScience.

[6] Brockerhoff EG, Barratt BIP, Beggs JR, Fagan LL, Kay N (2010). Impacts of exotic invertebrates on New Zealand's indigenous species and ecosystems.. New Zealand Journal of Ecology.

[7] Allen EA, Humble LM (2001). Nonindigenous species introductions: a threat to Canada's forests and forest economy.. Canadian Journal of Plant Pathology.

[8] Humble LM, Allen EA (2006). Forest biosecurity: alien invasive species and vectored organisms.. Canadian Journal of Plant Pathology.

[9] Haack RA (2006). Exotic bark- and wood-boring Coleoptera in the United States: recent establishments and interceptions.. Canadian Journal of Forest Research.

[10] Food and Agriculture Organization (FAO) (2006). International Standards for Phytosanitary Measures No. 27, Diagnostic protocols for regulated pests..

[11] Le Roux JJ, Wieczorek AM (2009). Molecular systematics and population genetics of biological invasions: towards a better understanding of invasive species management.. Annals of Applied Biology.

[12] Floyd R, Lima J, deWaard JR, Humble LR, Hanner RH (2010). Common goals: incorporating DNA barcoding into international protocols for identification of arthropod pests.. Biological Invasions. In press.

[13] Siddall ME, Budinoff RB (2005). DNA-barcoding evidence for widespread introductions of a leech from the South American *Helobdella triserialis* complex.. Conservation Genetics.

[14] Scheffer SJ, Lewis ML, Joshi RC (2006). DNA barcoding applied to invasive leafminers (Diptera: Agromyzidae) in the Philippines.. Annals of the Entomological Society of America.

[15] Armstrong KF, Ball SL (2005). DNA barcodes for biosecurity: invasive species identification.. Philosophical Transactions of the Royal Society B: Biological Sciences.

[16] Barr NB (2009). Pathway analysis of *Ceratitis capitata* (Diptera: Tephritidae) using mitochondrial DNA.. Journal of Economic Entomology.

[17] Smith MA, Fisher BL (2009). Invasions, DNA barcodes, and rapid biodiversity assessment using ants of Mauritius.. Frontiers in Zoology.

[18] Wilson AD, Schiff NM (2010). Identification of *Sirex noctilio* and native North American woodwasp larvae using DNA barcodes.. Journal of Entomology.

[19] Nadel RL, Slippers B, Scholes MC, Lawson SA, Noack AE (2010). DNA bar-coding reveals source and patterns of *Thaumastocoris peregrinus* invasions in South Africa and South America.. Biological Invasions.

[20] Simonsen TJ, Brown RL, Sperling FAH (2008). Tracing an invasion: phylogeography of *Cactoblastis cactorum* (Lepidoptera: Pyralidae) in the United States based on mitochondrial DNA.. Annals of the Entomological Society of America.

[21] Humble LM, deWaard JR, Quinn M (2009). Delayed recognition of the European poplar shoot borer, *Gypsonoma aceriana* (Duponchel) (Lepidoptera: Tortricidae) in Canada.. Journal of the Entomological Society of BC.

[22] deWaard JR, Landry J-F, Schmidt BC, Derhousoff J, McLean JA (2009). In the dark in a large urban park: DNA barcodes illuminate cryptic and introduced moth species.. Biodiversity and Conservation.

[23] Gilligan TM, Epstein ME (2009). LBAM ID, Tools for diagnosing light brown apple moth and related western U. S. leafrollers (Tortricidae: Archipini)..

[24] Bogdanowicz SM, Wallner WE, Bell J, Odell TM, Harrison RG (1993). Asian gypsy moths (Lepidoptera: Lymantriidae) in North America: evidence from molecular data.. Annals of the Entomological Society of America.

[25] Bogdanowicz SM, Mastro VC, Prasher DC, Harrison RG (1997). Microsatellite DNA variation among Asian and North American gypsy moths (Lepidoptera: Lymantriidae).. Annals of the Entomological Society of America.

[26] Bogdanowicz SM, Schaefer PW, Harrison RG (2000). Mitochondrial DNA variation among worldwide populations of gypsy moths, *Lymantria dispar*.. Molecular Phylogenetics and Evolution.

[27] Pfeifer TA, Humble LM, Ring M, Grigliatti TA (1995). Characterization of gypsy moth populations and related species using a nuclear DNA marker.. Canadian Entomology.

[28] Garner K, Slavicek JM (1996). Identification and characterization of a RAPD-PCR marker for distinguishing Asian and North American gypsy moths.. Insect Molecular Biology.

[29] Reineke A, Zebitz CW (1999). Suitability of polymerase chain reaction-based approaches for identification of different gypsy moth (Lepidoptera: Lymantriidae) genotypes in central Europe.. Annals of the Entomological Society of America.

[30] Koshio C, Tomishima M, Shimizu K, Kim H-S, Takenaka O (2002). Microsatellites in the gypsy moth, *Lymantria dispar* L. (Lepidoptera: Lymantriidae).. Applied Entomology and Zoology.

[31] Armstrong KF, McHugh P, Chinn W, Frampton ER, Walsh PJ (2003). Tussock moth species arriving on imported used vehicles determined by DNA analysis.. New Zealand Plant Protection.

[32] Keena MA, Cote M-J, Grinberg PS, Wallner WE (2008). World distribution of female flight and genetic variation in *Lymantria dispar* (Lepidoptera: Lymantriidae).. Environmental Entomology.

[33] Wallner WE, Humble LM, Levin RE, Baranchikov YN, Carde RT (1995). Response of adult lymantriid moths to illumination devices in the Russian Far East.. Journal of Economic Entomology.

[34] Pogue MG, Schaefer PW (2007). A review of selected species of *Lymantria* (Hübner [1819]) (Lepidoptera: Noctuidae: Lymantriinae) from subtropical and temperate regions of Asia including the description of three new species, some potentially invasive to North America..

[35] Ross MG (2005). Response to a gypsy moth incursion within New Zealand..

[36] Ball SL, Armstrong KF (2006). DNA barcodes for insect pest identification: a test case with tussock moths (Lepidoptera: Lymantriidae).. Canadian Journal of Forest Research.

[37] Schintlmeister A (2004). The taxonomy of the genus *Lymantria* Hübner, [1819] (Lepidoptera: Lymantriidae).. Quadrifina.

[38] Hebert PDN, deWaard JR, Landry J-F (2010). DNA barcodes for 1/1000 of the Animal Kingdom.. Biology Letters.

[39] Hajibabaei M, deWaard JR, Ivanova NV, Ratnasingham S, Dooh RT (2005). Critical factors for assembling a high volume of DNA barcodes.. Philosophical Transactions of the Royal Society B: Biological Sciences.

[40] deWaard JR, Ivanova NV, Hajibabaei M, Hebert PDN, Martin Cristofre (2008). Assembling DNA Barcodes: Analytical Protocols.. In Methods in Molecular Biology: Environmental Genetics.

[41] Hebert PDN, Penton EH, Burns JM, Janzen DH, Hallwachs W (2004). Ten species in one: DNA barcoding reveals cryptic species in the neotropical skipper butterfly *Astraptes fulgerator*.. Proceedings of the National Academy of Sciences of the United States of America.

[42] Hajibabaei M, Janzen DH, Burns JM, Hallwachs W, Hebert PDN (2006). DNA barcodes distinguish species of tropical Lepidoptera.. Proceedings of the National Academy of Sciences of the United States of America.

[43] Cho S, Mitchell A, Mitter C, Regier J, Matthews M (2008). Molecular phylogenetics of heliothine moths (Lepidoptera: Noctuidae: Heliothinae), with comments on the evolution of host range and pest status.. Systematic Entomology.

[44] Ratnasingham S, Hebert PDN (2007). http://www.barcodinglife.org.

[45] Zwickl DJ (2006). Genetic algorithm approaches for the phylogenetic analysis of large biological sequence datasets under the maximum likelihood criterion..

[46] Posada D, Crandall KA (1998). Modeltest: testing the model of DNA substitution.. Bioinformatics.

[47] Tamura K, Dudley J, Nei M, Kumar S (2007). MEGA4: Molecular Evolutionary Genetics Analysis (MEGA) software version 4.0.. Molecular Biology and Evolution.

[48] Kimura M (1980). A simple method for estimating evolutionary rate of base substitution through comparative studies of nucleotide sequences.. Journal of Molecular Evolution.

[49] Abdo Z, Golding GB (2007). A step toward barcoding life: a model-based, decision-theoretic method to assign genes to pre-existing species groups.. Systematic Biology.

[50] Lou M, Golding GB (2010). Assigning sequences to species in the absence of large interspecific differences.. Molecular Phylogenetics and Evolution.

[51] Tavares ES, Baker AJ (2008). Single mitochondrial gene barcodes reliably identify sister-species in diverse clades of birds.. BMC Evolutionary Biology.

[52] Weese DA, Santos SR (2009). Genetic identification of source populations for an aquarium-traded invertebrate.. Animal Conservation.

[53] Liebhold A, Mastro V, Schaefer PW (1989). Learning from the legacy of Leopold Trouvelot.. Bulletin of the Entomological Society of America.

[54] Bortolus A (2008). Error cascades in the biological sciences: The unwanted consequences of using bad taxonomy in ecology.. AMBIO: A Journal of the Human Environment.

