# Supplementary material for: Towards a Global Barcode Library for Lymantria (Lepidoptera: Lymantriinae) Tussock Moths of Biosecurity Concern
Source: PLoS One. 2010 Dec 9;5(12):e14280. doi: 10.1371/journal.pone.0014280 (PMC3000334; doi:10.1371/journal.pone.0014280)
Supplement: Figure S1 — Complete maximum likelihood tree for 36 species of Lymantria. Full tree from Figure 3 displaying haplotype variation within species clusters. BOLD process IDs and collection localities are provided for each sequence. Tree was rooted with the outgroup Orgyia antiqua. (0.02 MB PDF) [file pone.0014280.s002.pdf]

Appendix S1. Taxonomic treatment of *Lymantria nebulosa* Wileman, revised status and *Lymantria subpallida* Okano, revised status

The following taxonomic changes are presented in light of the DNA barcoding results of the present study and subsequent morphological investigation.

***Lymantria* (*Lymantria*) *nebulosa* Wileman, revised status**

(Figs. 1, 5)

Lymantria nebulosa Wileman, 1910:309. Holotype male: Taiwan: (BMNH: London, examined); Strand, 1923:324; Bang-Haas, 1926:132; Bryk, 1934:166; Schintlmeister, 2004:112; Pogue and Schaefer, 2007:97.

Lymantria nebulosa forma *aprilis* Strand, 1923:324. Holotype male: Taiwan (ZMHB, Berlin, not examined); Bang-Haas, 1926:132; Bryk, 1934:166; Pogue and Schaefer, 2007:97.

Discussion. Schintlmeister (2004) synonymized *L. nebulosa* with *L. sinica*. The results of the present study indicated that *L. nebulosa* was sufficiently distinct from *L. sinica* to warrant its status as a valid species. The following morphological differences support these molecular results.

The adult male specimens are similar in size, pattern, and overall coloration. The male of *L. nebulosa* (Fig. 1) is worn, so the pattern is faint and hard to diagnose it from *L. sinica* (Fig. 2). *Lymantria sinica* is much darker and has a distinct, white, zig-zag postmedial line, this line is not evident in *L.*

nebulosa due to the poor condition of the specimen. Schintlmeister (2004) states that *L. sinica* is quite variable from specimens having a white ground color to those being very dark as in Fig. 1. The male genitalia of the holotype of *L. sinica* is illustrated in Fig. 6. The principle differences between *L. sinica* and *L. nebulosa* are in structures of the valve. The apex of the valve is produced into a slightly elongate projection. In *L. sinica* the apical projection is narrow and along the same plane as the costa of the valve (Fig. 6). In *L. nebulosa* the apical projection is upright and broader at the base (Fig. 5). The ampulla is an elongate, median projection from the valve with a pointed, decurved apex and a vertical spine near its base. In *L. sinica* the vertical spine is more than twice the size as in *L. nebulosa*. The ventral distal angle of the valve is slightly produced in *L. sinica* and not produced in *L. nebulosa*. The aedeagus is very slightly sinuate in *L. sinica* and in *L. nebulosa* it is distinctly bent. The vesica is oval shaped in *L. sinica* and its long axis is parallel to the aedeagus. In *L. nebulosa* the vesica is oblong and emerges from the aedeagus with its long axis a right angle to the aedeagus.

***Lymantria* (*Nyctria*) *subpallida* Okano, revised status**

(Figs. 3, 7)

Lymantria mathura subpallida Okano, 1960:36. Holotype male: Taiwan: Puli-Wushe (Okano collection, not examined); Schintlmeister, 2004:127; Pogue and Schaefer, 2007:112.

Discusssion. Schintlmeister (2004) synonymized *L. m. subpallida* with *L. m. aurora* Butler, stating that Okano (1960) gave no features in which to separate it from *L. m. aurora.* The results of this study clearly show that *L. subpallida* is molecularly distinct from *L. mathura* Moore. The following morphological differences support these molecular results.

There are virtually no differences in the overall pattern and color of *L. subpallida* (Fig. 3) and *L. mathura* (Fig. 4). The male genitalia exhibit several differences. The tegumen is more oval, being slightly higher vertically than horizontally in *L. mathura* (Fig. 8), and more round in *L. subpallida* (Fig. 7). The lateral process of the tegumen is much broader at the base in *L. mathura* than it is in *L. subpallida*. The valve is divided with the dorsal process bifurcate. In *L. mathura* the dorsal bifurcation is large and in *L. subpallida* the dorsal bifurcation is small, less than half the size as in *L. mathura*. The relative length of the ventral process of the valve to the overall length of the dorsal process in different between these species. In *L. mathura* the length of the ventral process is 2/3 the overall length of the dorsal process and in *L. subpallida* the ventral process in 3/4 the overall length.

References

Bang-Haas, O. 1926. Novitates macrolepidoterologicae: Katalog der im “Seitz” nicht enthalten und seitdem neu beschriebenen palaearkitschen Macrolepidopteren // herausgegeben von Otto Bang-Haas. Blasewitz, Dresden, 238 pp.

Bryk, F. 1934. Pars 62: Lymantriidae. *In*: Strand, E. (ed.) Lepidoptorum Catalogus. W. Junk, Berlin, 441 pp.

Okano, M. 1960. New of little known moths from Formosa (3). Annual Report of the Gagukei Faculty of the Iwate University 15: 35–40.

Pogue, M.G. and P.W. Schaefer. 2007. A review of selected species of *Lymantria* Hübner [1819] including three new species (Lepidoptera: Noctuidae: Lymantriinae). Forest Health Technology Enterprise Team, Technology Transfer, FHTET-2006-07, 223 pp.

Schintlmeister, A. 2004. The taxonomy of the genus *Lymantria* Hübner [1819] (Lepidoptera: Lymantriidae). Quadrifina 7:1–248.

Strand, E. 1923. 30. Genus: *Lymantria*. pp. 321–328. *In:* Seitz, A. (ed.). The Macrolepidoptera of the World. 10. Volume: The Indo-Australian Bombyces and Sphinges. Alfred Kernen, Stuttgart. 909 pp., 104 plates. [Signature 40, published 30 October 1923].

Wileman, A.E. 1910. New Lepidoptera Heterocera from Formosa. The Entomologist 43:309–313.

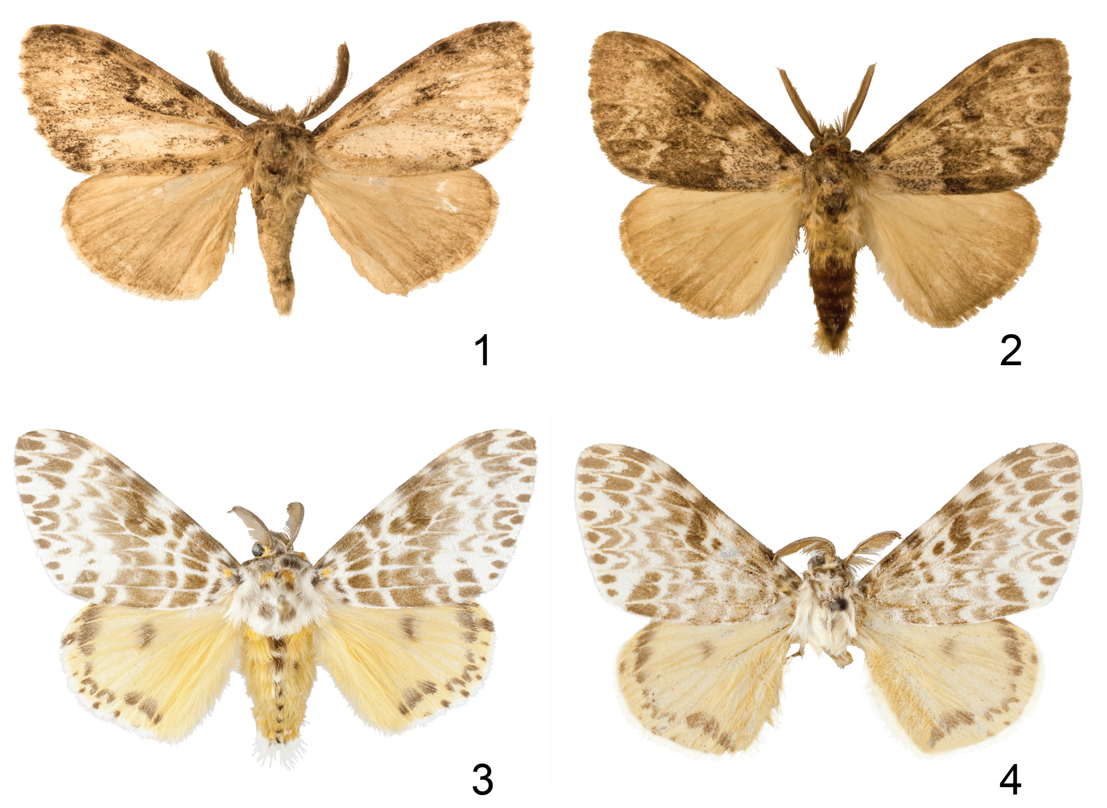

Figs. 1–4. Adults. 1. *Lymantria nebulosa*, China, Hainan, Wuzhi Shan. 2. *Lymantria sinica*, China, Hong Kong, Kadoori [sic] Agricultural Research Centre. 3. *Lymantria subpallida*, Taiwan, Nan Tow Co., Lu-shan, ca. 30 km E Wu-she. 4. *Lymantria mathura*, China, Yunnan.

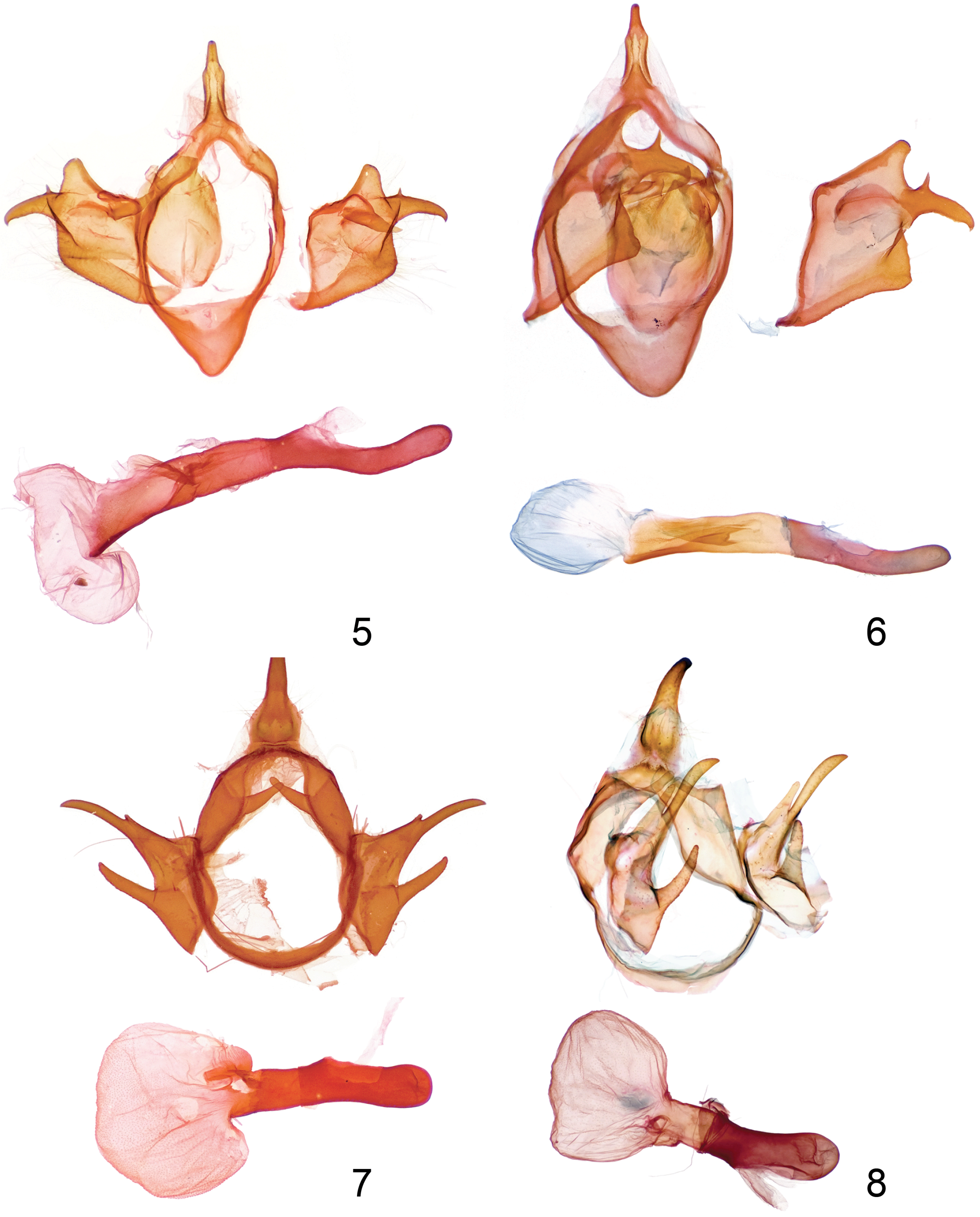

Figs. 5–8. Male genitalia. 5. *Lymantria nebulosa*, China, Hainan, Wuzhi Shan. 6. *Lymantria sinica*, Holotype, N. China. 7. *Lymantria subpallida*, Hong, Kadoorie [sic] Agricultural Research Centre. 8. *Lymantria mathura*, Ceylon [Sir Lanka].
