# Supplementary material for: Towards a Global Barcode Library for Lymantria (Lepidoptera: Lymantriinae) Tussock Moths of Biosecurity Concern
Source: PLoS One. 2010 Dec 9;5(12):e14280. doi: 10.1371/journal.pone.0014280 (PMC3000334; doi:10.1371/journal.pone.0014280)
Supplement: Table S1 — Taxonomic samples used in study, including Barcode of Life Database (BOLD) and GenBank accessions. Also listed is the presence (+) or absence (-) of the NlaIII (N) and BamHI (B) restriction enzyme sites of the ‘NB system’ [24], determined from the sequence data (i.e. not by performing the assay). (0.16 MB PDF) [file pone.0014280.s003.pdf]

**Table S1.** Taxonomic samples used in study, including Barcode of Life Database (BOLD) and GenBank accessions. Also listed is the presence (+) or absence (-) of the NlaIII (N) and BamHI (B) restriction enzyme sites of the 'NB system' [24], determined from the sequence data (i.e. not by performing the assay).

| Identification | BOLD ID | Specimen ID | Genbank No. | Institution Storing | Country | Locality | Publication | NB Haplotype | |
|---|---|---|---|---|---|---|---|---|---|
| *Lymantria albescens* | BOGDA065-08 | Bogda-JA11-65 | N/A[1] | Unknown (GenBank, NCBI) | Japan | Okinawa | Bogdanowicz et al. 2000 | N+ | B- |
| *Lymantria albescens* | GBGL4530-07 | AF075274 | AF075274 | Unknown (GenBank, NCBI) | Japan | Okinawa | Bogdanowicz et al. 2000 | N+ | B- |
| *Lymantria albescens* | LYMAN031-08 | ww01229 | HM775520 | Smithsonian National Museum of Natural History | Japan | Okinawa | Present study | N+ | N/A |
| *Lymantria albescens* | LYMAN032-08 | ww01230 | HM775519 | Smithsonian National Museum of Natural History | Japan | Okinawa | Present study | N+ | B- |
| *Lymantria albescens* | LYMAN033-08 | ww01231 | HM775518 | Smithsonian National Museum of Natural History | Japan | Okinawa | Present study | N+ | N/A |
| *Lymantria albescens* | LYMAN034-08 | ww01232 | HM775517 | Smithsonian National Museum of Natural History | Japan | Okinawa | Present study | N+ | B- |
| *Lymantria albescens* | LYMAN035-08 | ww01233 | HM775516 | Smithsonian National Museum of Natural History | Japan | Okinawa | Present study | N+ | B- |
| *Lymantria albescens* | LYMAN036-08 | ww01234 | HM775515 | Smithsonian National Museum of Natural History | Japan | Okinawa | Present study | N+ | B- |
| *Lymantria albescens* | LYMAN037-08 | ww01235 | HM775514 | Smithsonian National Museum of Natural History | Japan | Okinawa | Present study | N+ | B- |
| *Lymantria albescens* | LYMAN038-08 | ww01236 | HM775513 | Smithsonian National Museum of Natural History | Japan | Okinawa | Present study | N+ | B- |
| *Lymantria albescens* | LYMAN039-08 | ww01237 | HM775512 | Smithsonian National Museum of Natural History | Japan | Okinawa | Present study | N+ | B- |
| *Lymantria albescens* | LYMAN076-08 | ww01274 | HM775511 | Smithsonian National Museum of Natural History | Japan | Okinawa | Present study | N+ | B- |
| *Lymantria albescens* | LYMAN077-08 | ww01275 | HM775510 | Smithsonian National Museum of Natural History | Japan | Okinawa | Present study | N+ | N/A |
| *Lymantria antennata* | GBGL1614-06 | Lepi477 | DQ149570 | Unknown (GenBank, NCBI) | Australia | Queensland | Ball & Armstrong 2006 | N- | B- |
| *Lymantria antennata* | GBGL1615-06 | Lepi475 | DQ149571 | Unknown (GenBank, NCBI) | Australia | Queensland | Ball & Armstrong 2006 | N- | B- |
| *Lymantria antennata* | GBGL1616-06 | Lepi474 | DQ149572 | Unknown (GenBank, NCBI) | Australia | Queensland | Ball & Armstrong 2006 | N- | B- |
| *Lymantria antennata* | LYMAN162-08 | ww02142 | HM775522 | Agricultural Scientific Collections Unit, NSW | Australia | New South Wales | Present study | N- | N/A |
| *Lymantria antennata* | LYMAN164-08 | ww02144 | HM775521 | Agricultural Scientific Collections Unit, NSW | Australia | New South Wales | Present study | N- | B- |
| *Lymantria antennata* | LYMAN165-08 | ww02145 | HM775529 | Agricultural Scientific Collections Unit, NSW | Australia | New South Wales | Present study | N- | N/A |
| *Lymantria antennata* | LYMAN166-08 | ww02244 | HM775528 | Wagga Wagga Agricultural Institute | Australia | Queensland | Present study | N- | N/A |
| *Lymantria antennata* | LYMAN167-08 | ww02245 | HM775527 | Wagga Wagga Agricultural Institute | Australia | Queensland | Present study | N- | N/A |
| *Lymantria antennata* | LYMAN168-08 | ww02246 | HM775526 | Wagga Wagga Agricultural Institute | Australia | Queensland | Present study | N- | B- |
| *Lymantria antennata* | LYMAN170-08 | ww02248 | HM775525 | Wagga Wagga Agricultural Institute | Australia | Queensland | Present study | N- | B- |
| *Lymantria antennata* | LYMAN171-08 | ww02249 | HM775524 | Wagga Wagga Agricultural Institute | Australia | Queensland | Present study | N- | B- |
| *Lymantria antennata* | LYMAN172-08 | ww02250 | HM775523 | Wagga Wagga Agricultural Institute | Australia | Queensland | Present study | N- | B- |
| *Lymantria antennata* | LYMAN173-08 | ww02251 | HM775531 | Wagga Wagga Agricultural Institute | Australia | Queensland | Present study | N- | B- |
| *Lymantria antennata* | LYMAN174-08 | ww02252 | HM775530 | Wagga Wagga Agricultural Institute | Australia | Queensland | Present study | N- | B- |
| *Lymantria atemeles* | GBGL1601-06 | Lepi453 | DQ116184 | Unknown (GenBank, NCBI) | Thailand | Kampaeng Saen | Armstrong & Ball 2005 | N- | B- |
| *Lymantria atemeles* | LYMAN040-08 | ww01238 | HM775534 | Smithsonian National Museum of Natural History | Thailand | Kampaeng Saen | Present study | N- | N/A |
| *Lymantria atemeles* | LYMAN041-08 | ww01239 | HM775533 | Smithsonian National Museum of Natural History | Thailand | Kampaeng Saen | Present study | N- | N/A |
| *Lymantria atemeles* | LYMAN042-08 | ww01240 | HM775532 | Smithsonian National Museum of Natural History | Thailand | Kampaeng Saen | Present study | N- | N/A |
| *Lymantria atemeles* | LYMAN175-08 | ww02253 | HM775535 | Wagga Wagga Agricultural Institute | Thailand | Chantabury | Present study | N- | N/A |
| *Lymantria bantaizana* | GBGL1580-06 | Ly230 | DQ116163 | Unknown (GenBank, NCBI) | Japan | Honshu | Armstrong & Ball 2005 | N+ | B- |
| *Lymantria bantaizana* | GBGL1585-06 | Ly231 | DQ116168 | Unknown (GenBank, NCBI) | Japan | Honshu | Armstrong & Ball 2005 | N+ | B- |
| *Lymantria bantaizana* | GBGL1590-06 | Ly232 | DQ116173 | Unknown (GenBank, NCBI) | Japan | Honshu | Armstrong & Ball 2005 | N+ | B- |
| *Lymantria bantaizana* | GBGL1595-06 | Ly228 | DQ116178 | Unknown (GenBank, NCBI) | Japan | Honshu | Armstrong & Ball 2005 | N+ | B- |
| *Lymantria brunneiplaga* | LTOLB207-09 | AYK-04-0840-03 | HM775536 | University of Maryland | Malaysia | | Present study | N+ | B- |
| *Lymantria concolor* | LYMAN081-08 | ww01279 | HM775537 | Smithsonian National Museum of Natural History | Nepal | Jiri | Present study | N+ | N/A |
| *Lymantria dispar* | BOGDA045-08 | Bogda-JA5-45 | N/A[1] | Unknown (GenBank, NCBI) | Japan | Oshima Island | Bogdanowicz et al. 2000 | N+ | B+ |
| *Lymantria dispar* | BOGDA046-08 | Bogda-JA5-46 | N/A[1] | Unknown (GenBank, NCBI) | Japan | Ibaraki Pref. | Bogdanowicz et al. 2000 | N+ | B+ |
| *Lymantria dispar* | BOGDA047-08 | Bogda-JA5-47 | N/A[1] | Unknown (GenBank, NCBI) | Japan | Ibaraki Pref. | Bogdanowicz et al. 2000 | N+ | B+ |
| *Lymantria dispar* | BOGDA048-08 | Bogda-JA5-48 | N/A[1] | Unknown (GenBank, NCBI) | Japan | Ibaraki Pref. | Bogdanowicz et al. 2000 | N+ | B+ |
| *Lymantria dispar* | BOGDA049-08 | Bogda-JA5-49 | N/A[1] | Unknown (GenBank, NCBI) | Japan | Ibaraki Pref. | Bogdanowicz et al. 2000 | N+ | B+ |
| *Lymantria dispar* | BOGDA050-08 | Bogda-JA5-50 | N/A[1] | Unknown (GenBank, NCBI) | Japan | Ibaraki Pref. | Bogdanowicz et al. 2000 | N+ | B+ |
| *Lymantria dispar* | BOGDA051-08 | Bogda-JA5-51 | N/A[1] | Unknown (GenBank, NCBI) | Japan | Ibaraki Pref. | Bogdanowicz et al. 2000 | N+ | B+ |
| *Lymantria dispar* | BOGDA064-08 | Bogda-JA10-64 | N/A[1] | Unknown (GenBank, NCBI) | Japan | Hokkaido | Bogdanowicz et al. 2000 | N+ | B+ |
| *Lymantria dispar* | GBGL1529-06 | Ly35 | DQ116112 | Unknown (GenBank, NCBI) | Japan | | Armstrong & Ball 2005 | N+ | B+ |
| *Lymantria dispar* | GBGL1536-06 | Ly33 | DQ116119 | Unknown (GenBank, NCBI) | Japan | | Armstrong & Ball 2005 | N+ | B+ |
| *Lymantria dispar* | GBGL1537-06 | Ly34 | DQ116120 | Unknown (GenBank, NCBI) | Japan | | Armstrong & Ball 2005 | N+ | N/A |
| *Lymantria dispar* | GBGL1538-06 | Ly41 | DQ116121 | Unknown (GenBank, NCBI) | Japan | | Armstrong & Ball 2005 | N+ | B+ |
| *Lymantria dispar* | GBGL4430-07 | AB244647 | AB244647 | Unknown (GenBank, NCBI) | Japan | Hokkaido | Yamaguchi et al. unpublished | N+ | B+ |
| *Lymantria dispar* | LBCH7981-10 | 10-JDWBC-7981 | HM775700 | Canadian National Collection | Canada[2] | British Columbia | Present study | N+ | B+ |
| *Lymantria dispar* | LYMAN045-08 | ww01243 | HM775704 | Smithsonian National Museum of Natural History | Kyrgyzstan | Karalma | Present study | N+ | N/A |
| *Lymantria dispar* | LYMAN046-08 | ww01244 | HM775703 | Smithsonian National Museum of Natural History | Kyrgyzstan | Karalma | Present study | N+ | B- |
| *Lymantria dispar* | LYMAN047-08 | ww01245 | HM775702 | Smithsonian National Museum of Natural History | Kyrgyzstan | Toktogul | Present study | N+ | N/A |
| *Lymantria dispar* | LYMAN048-08 | ww01246 | HM775701 | Smithsonian National Museum of Natural History | Kyrgyzstan | Toktogul | Present study | N+ | B- |
| *Lymantria dispar* | LYMMK112-09 | LymMk_RB-1 | HM775591 | USDA Forest Service, Northern Research Station | Russia | Krasnojarsk | Present study | N+ | B- |
| *Lymantria dispar* | LYMMK113-09 | LymMk_RB-2 | HM775590 | USDA Forest Service, Northern Research Station | Russia | Krasnojarsk | Present study | N+ | B- |
| *Lymantria dispar* | LYMMK114-09 | LymMk_RB-3 | HM775589 | USDA Forest Service, Northern Research Station | Russia | Krasnojarsk | Present study | N+ | B- |
| *Lymantria dispar* | LYMMK115-09 | LymMk_RBI-1 | HM775588 | USDA Forest Service, Northern Research Station | Russia | Khakassia | Present study | N+ | B- |
| *Lymantria dispar* | LYMMK116-09 | LymMk_RBI-2 | HM775586 | USDA Forest Service, Northern Research Station | Russia | Khakassia | Present study | N+ | B- |
| *Lymantria dispar* | LYMMK117-09 | LymMk_RBI-3 | HM775585 | USDA Forest Service, Northern Research Station | Russia | Khakassia | Present study | N+ | B- |
| *Lymantria dispar asiatica* | BOGDA016-08 | Bogda-RA2-16 | N/A[1] | Unknown (GenBank, NCBI) | Russia | Irkutskaya Oblast' | Bogdanowicz et al. 2000 | N+ | B+ |
| *Lymantria dispar asiatica* | BOGDA017-08 | Bogda-RA2-17 | N/A[1] | Unknown (GenBank, NCBI) | Russia | Irkutskaya Oblast' | Bogdanowicz et al. 2000 | N+ | B+ |
| *Lymantria dispar asiatica* | BOGDA018-08 | Bogda-RA3-18 | N/A[1] | Unknown (GenBank, NCBI) | Russia | Primorskiy Kray | Bogdanowicz et al. 2000 | N+ | B+ |
| *Lymantria dispar asiatica* | BOGDA019-08 | Bogda-RA3-19 | N/A[1] | Unknown (GenBank, NCBI) | Russia | Primorskiy Kray | Bogdanowicz et al. 2000 | N+ | B+ |
| *Lymantria dispar asiatica* | BOGDA020-08 | Bogda-RA3-20 | N/A[1] | Unknown (GenBank, NCBI) | Russia | Primorskiy Kray | Bogdanowicz et al. 2000 | N+ | B+ |
| *Lymantria dispar asiatica* | BOGDA021-08 | Bogda-RA3-21 | N/A[1] | Unknown (GenBank, NCBI) | Russia | Primorskiy Kray | Bogdanowicz et al. 2000 | N+ | B+ |
| *Lymantria dispar asiatica* | BOGDA022-08 | Bogda-RA3-22 | N/A[1] | Unknown (GenBank, NCBI) | Russia | Primorskiy Kray | Bogdanowicz et al. 2000 | N- | B- |
| *Lymantria dispar asiatica* | BOGDA023-08 | Bogda-RA3-23 | N/A[1] | Unknown (GenBank, NCBI) | Russia | Primorskiy Kray | Bogdanowicz et al. 2000 | N+ | B+ |
| *Lymantria dispar asiatica* | BOGDA024-08 | Bogda-RA3-24 | N/A[1] | Unknown (GenBank, NCBI) | Russia | Primorskiy Kray | Bogdanowicz et al. 2000 | N+ | B+ |
| *Lymantria dispar asiatica* | BOGDA025-08 | Bogda-RA3-25 | N/A[1] | Unknown (GenBank, NCBI) | Russia | Primorskiy Kray | Bogdanowicz et al. 2000 | N+ | B+ |
| *Lymantria dispar asiatica* | BOGDA026-08 | Bogda-RA4-26 | N/A[1] | Unknown (GenBank, NCBI) | Russia | Khabarovskiy Kray | Bogdanowicz et al. 2000 | N+ | B+ |
| *Lymantria dispar asiatica* | BOGDA027-08 | Bogda-RA4-27 | N/A[1] | Unknown (GenBank, NCBI) | Russia | Khabarovskiy Kray | Bogdanowicz et al. 2000 | N+ | B+ |
| *Lymantria dispar asiatica* | BOGDA028-08 | Bogda-CH1-28 | N/A[1] | Unknown (GenBank, NCBI) | China | Heilongjiang Sheng | Bogdanowicz et al. 2000 | N+ | B+ |
| *Lymantria dispar asiatica* | BOGDA029-08 | Bogda-CH1-29 | N/A[1] | Unknown (GenBank, NCBI) | China | Heilongjiang Sheng | Bogdanowicz et al. 2000 | N+ | B+ |
| *Lymantria dispar asiatica* | BOGDA030-08 | Bogda-CH2-30 | N/A[1] | Unknown (GenBank, NCBI) | China | Liaoning | Bogdanowicz et al. 2000 | N+ | B+ |

| | | | | | | | | | |
|---|---|---|---|---|---|---|---|---|---|
| *Lymantria dispar asiatica* | BOGDA031-08 | Bogda-CH2-31 | N/A[1] | Unknown (GenBank, NCBI) | China | Liaoning | Bogdanowicz et al. 2000 | N+ | B+ |
| *Lymantria dispar asiatica* | BOGDA032-08 | Bogda-CH3-32 | N/A[1] | Unknown (GenBank, NCBI) | China | Hebei | Bogdanowicz et al. 2000 | N+ | B+ |
| *Lymantria dispar asiatica* | BOGDA033-08 | Bogda-CH3-33 | N/A[1] | Unknown (GenBank, NCBI) | China | Hebei | Bogdanowicz et al. 2000 | N+ | B+ |
| *Lymantria dispar asiatica* | BOGDA034-08 | Bogda-CH4-34 | N/A[1] | Unknown (GenBank, NCBI) | China | Beijing Shi | Bogdanowicz et al. 2000 | N+ | B+ |
| *Lymantria dispar asiatica* | BOGDA035-08 | Bogda-CH4-35 | N/A[1] | Unknown (GenBank, NCBI) | China | Beijing Shi | Bogdanowicz et al. 2000 | N+ | B+ |
| *Lymantria dispar asiatica* | BOGDA036-08 | Bogda-KOR-36 | N/A[1] | Unknown (GenBank, NCBI) | South Korea | Seoul | Bogdanowicz et al. 2000 | N+ | B+ |
| *Lymantria dispar asiatica* | BOGDA037-08 | Bogda-KOR-37 | N/A[1] | Unknown (GenBank, NCBI) | South Korea | Seoul | Bogdanowicz et al. 2000 | N+ | B+ |
| *Lymantria dispar asiatica* | GBGL1523-06 | Ly1L | DQ116106 | Unknown (GenBank, NCBI) | Russia | Sakhalinskaya Oblast | Armstrong & Ball 2005 | N+ | B+ |
| *Lymantria dispar asiatica* | GBGL1524-06 | GP2 | DQ116107 | Unknown (GenBank, NCBI) | Russia | Sakhalinskaya Oblast | Armstrong & Ball 2005 | N+ | B+ |
| *Lymantria dispar asiatica* | GBGL1567-06 | Ly218 | DQ116150 | Unknown (GenBank, NCBI) | South Korea | Wontong | Armstrong & Ball 2005 | N+ | N/A |
| *Lymantria dispar asiatica* | GBGL1568-06 | Ly219 | DQ116151 | Unknown (GenBank, NCBI) | South Korea | Wontong | Armstrong & Ball 2005 | N+ | B+ |
| *Lymantria dispar asiatica* | GBGL1569-06 | Ly220 | DQ116152 | Unknown (GenBank, NCBI) | South Korea | Wontong | Armstrong & Ball 2005 | N+ | B+ |
| *Lymantria dispar asiatica* | GBGL1586-06 | Ly222 | DQ116169 | Unknown (GenBank, NCBI) | South Korea | Wontong | Armstrong & Ball 2005 | N+ | B+ |
| *Lymantria dispar asiatica* | LYMAN011-08 | ww01209 | HM775708 | Smithsonian National Museum of Natural History | Mongolia | Ulaanba-atar | Present study | N+ | B+ |
| *Lymantria dispar asiatica* | LYMAN012-08 | ww01210 | HM775707 | Smithsonian National Museum of Natural History | Mongolia | Ulaanba-atar | Present study | N+ | N/A |
| *Lymantria dispar asiatica* | LYMAN013-08 | ww01211 | HM775706 | Smithsonian National Museum of Natural History | China | Liaoning | Present study | N+ | N/A |
| *Lymantria dispar asiatica* | LYMAN014-08 | ww01212 | HM775705 | Smithsonian National Museum of Natural History | China | Liaoning | Present study | N+ | N/A |
| *Lymantria dispar asiatica* | LYMMK013-09 | LymMk_AA-1 | HM775695 | USDA Forest Service, Northern Research Station | Canada[2] | British Columbia | Present study | N+ | B+ |
| *Lymantria dispar asiatica* | LYMMK014-09 | LymMk_AA-2 | HM775694 | USDA Forest Service, Northern Research Station | Canada[2] | British Columbia | Present study | N+ | B+ |
| *Lymantria dispar asiatica* | LYMMK016-09 | LymMk_CB-1 | HM775692 | USDA Forest Service, Northern Research Station | China | Beijing Shi | Present study | N+ | B+ |
| *Lymantria dispar asiatica* | LYMMK017-09 | LymMk_CB-2 | HM775691 | USDA Forest Service, Northern Research Station | China | Beijing Shi | Present study | N+ | B+ |
| *Lymantria dispar asiatica* | LYMMK018-09 | LymMk_CB-3 | HM775690 | USDA Forest Service, Northern Research Station | China | Beijing Shi | Present study | N+ | B+ |
| *Lymantria dispar asiatica* | LYMMK019-09 | LymMk_CH-1 | HM775689 | USDA Forest Service, Northern Research Station | China | Hebei | Present study | N+ | B+ |
| *Lymantria dispar asiatica* | LYMMK020-09 | LymMk_CH-2 | HM775688 | USDA Forest Service, Northern Research Station | China | Hebei | Present study | N+ | B+ |
| *Lymantria dispar asiatica* | LYMMK021-09 | LymMk_CH-3 | HM775687 | USDA Forest Service, Northern Research Station | China | Hebei | Present study | N+ | B+ |
| *Lymantria dispar asiatica* | LYMMK022-09 | LymMk_CL-1 | HM775686 | USDA Forest Service, Northern Research Station | China | Liaoning | Present study | N+ | B+ |
| *Lymantria dispar asiatica* | LYMMK023-09 | LymMk_CL-2 | HM775608 | USDA Forest Service, Northern Research Station | China | Liaoning | Present study | N+ | B+ |
| *Lymantria dispar asiatica* | LYMMK024-09 | LymMk_CL-3 | HM775607 | USDA Forest Service, Northern Research Station | China | Liaoning | Present study | N+ | B+ |
| *Lymantria dispar asiatica* | LYMMK025-09 | LymMk_CS-1 | HM775685 | USDA Forest Service, Northern Research Station | China | Shandong | Present study | N+ | B+ |
| *Lymantria dispar asiatica* | LYMMK026-09 | LymMk_CS-2 | HM775605 | USDA Forest Service, Northern Research Station | China | Shandong | Present study | N+ | B+ |
| *Lymantria dispar asiatica* | LYMMK027-09 | LymMk_CS-3 | HM775684 | USDA Forest Service, Northern Research Station | China | Shandong | Present study | N+ | B+ |
| *Lymantria dispar asiatica* | LYMMK118-09 | LymMk_RM-1 | HM775584 | USDA Forest Service, Northern Research Station | Russia | Primorskiy Kray | Present study | N+ | B+ |
| *Lymantria dispar asiatica* | LYMMK119-09 | LymMk_RM-2 | HM775583 | USDA Forest Service, Northern Research Station | Russia | Primorskiy Kray | Present study | N+ | B+ |
| *Lymantria dispar asiatica* | LYMMK120-09 | LymMk_RM-3 | HM775582 | USDA Forest Service, Northern Research Station | Russia | Primorskiy Kray | Present study | N+ | B+ |
| *Lymantria dispar asiatica* | RFELP029-08 | PaA-08-552 | HM775618 | Pacific Forestry Centre, Canadian Forest Service | Russia | Primorskiy Kray | Present study | N+ | B+ |
| *Lymantria dispar asiatica* | RFELP030-08 | PaA-08-553 | HM775617 | Pacific Forestry Centre, Canadian Forest Service | Russia | Primorskiy Kray | Present study | N+ | B+ |
| *Lymantria dispar asiatica* | RFELP031-08 | PaA-08-554 | HM775616 | Pacific Forestry Centre, Canadian Forest Service | Russia | Primorskiy Kray | Present study | N+ | N/A |
| *Lymantria dispar asiatica* | RFELP032-08 | PaA-08-555 | HM775615 | Pacific Forestry Centre, Canadian Forest Service | Russia | Primorskiy Kray | Present study | N+ | B+ |
| *Lymantria dispar asiatica* | RFELP033-08 | PaA-08-556 | HM775614 | Pacific Forestry Centre, Canadian Forest Service | Russia | Primorskiy Kray | Present study | N+ | N/A |
| *Lymantria dispar dispar* | BOGDA001-08 | Bogda-US1-01 | N/A[1] | Unknown (GenBank, NCBI) | United States | New Jersey | Bogdanowicz et al. 2000 | N- | B- |
| *Lymantria dispar dispar* | BOGDA002-08 | Bogda-US2-02 | N/A[1] | Unknown (GenBank, NCBI) | United States | Vermont | Bogdanowicz et al. 2000 | N- | B- |
| *Lymantria dispar dispar* | BOGDA003-08 | Bogda-CAN-03 | N/A[1] | Unknown (GenBank, NCBI) | Canada | Ontario | Bogdanowicz et al. 2000 | N- | B- |
| *Lymantria dispar dispar* | BOGDA004-08 | Bogda-CAN-04 | N/A[1] | Unknown (GenBank, NCBI) | Canada | Ontario | Bogdanowicz et al. 2000 | N- | B- |
| *Lymantria dispar dispar* | BOGDA005-08 | Bogda-FRA-05 | N/A[1] | Unknown (GenBank, NCBI) | France | Provence-Alpes-Cote d`Azur | Bogdanowicz et al. 2000 | N+ | B- |
| *Lymantria dispar dispar* | BOGDA006-08 | Bogda-FRA-06 | N/A[1] | Unknown (GenBank, NCBI) | France | Provence-Alpes-Cote d`Azur | Bogdanowicz et al. 2000 | N+ | B- |
| *Lymantria dispar dispar* | BOGDA007-08 | Bogda-GER-07 | N/A[1] | Unknown (GenBank, NCBI) | Germany | Baden-Wuerttemberg | Bogdanowicz et al. 2000 | N+ | B- |
| *Lymantria dispar dispar* | BOGDA008-08 | Bogda-GER-08 | N/A[1] | Unknown (GenBank, NCBI) | Germany | Baden-Wuerttemberg | Bogdanowicz et al. 2000 | N+ | B- |
| *Lymantria dispar dispar* | BOGDA009-08 | Bogda-SAR-09 | N/A[1] | Unknown (GenBank, NCBI) | Italy | Sardinia | Bogdanowicz et al. 2000 | N+ | B- |
| *Lymantria dispar dispar* | BOGDA010-08 | Bogda-SLO-10 | N/A[1] | Unknown (GenBank, NCBI) | Slovakia | Kurinec | Bogdanowicz et al. 2000 | N+ | B- |
| *Lymantria dispar dispar* | BOGDA011-08 | Bogda-SLO-11 | N/A[1] | Unknown (GenBank, NCBI) | Slovakia | Kurinec | Bogdanowicz et al. 2000 | N+ | B- |
| *Lymantria dispar dispar* | BOGDA012-08 | Bogda-RA1-12 | N/A[1] | Unknown (GenBank, NCBI) | Russia | Moscow City | Bogdanowicz et al. 2000 | N+ | B- |
| *Lymantria dispar dispar* | BOGDA013-08 | Bogda-RA1-13 | N/A[1] | Unknown (GenBank, NCBI) | Russia | Moscow City | Bogdanowicz et al. 2000 | N+ | B- |
| *Lymantria dispar dispar* | BOGDA014-08 | Bogda-TUN-14 | N/A[1] | Unknown (GenBank, NCBI) | Tunisia | Jendouba | Bogdanowicz et al. 2000 | N+ | B- |
| *Lymantria dispar dispar* | BOGDA015-08 | Bogda-TUN-15 | N/A[1] | Unknown (GenBank, NCBI) | Tunisia | Jendouba | Bogdanowicz et al. 2000 | N+ | B- |
| *Lymantria dispar dispar* | GBGL1515-06 | Ly23 | DQ116098 | Unknown (GenBank, NCBI) | Canada | Ontario | Armstrong & Ball 2005 | N- | B- |
| *Lymantria dispar dispar* | GBGL1520-06 | Ly20 | DQ116103 | Unknown (GenBank, NCBI) | Canada | Ontario | Armstrong & Ball 2005 | N- | B- |
| *Lymantria dispar dispar* | GBGL1535-06 | Ly21 | DQ116118 | Unknown (GenBank, NCBI) | Canada | Ontario | Armstrong & Ball 2005 | N- | B- |
| *Lymantria dispar dispar* | GBGL1579-06 | Ly161 | DQ116162 | Unknown (GenBank, NCBI) | United States | West Virginia | Armstrong & Ball 2005 | N- | B- |
| *Lymantria dispar dispar* | GBGL1602-06 | Lepi454 | DQ116185 | Unknown (GenBank, NCBI) | United States | | Armstrong & Ball 2005 | N- | B- |
| *Lymantria dispar dispar* | GBGL4532-07 | AF075272 | AF075272 | Unknown (GenBank, NCBI) | United States | | Bogdanowicz et al. 2000 | N- | B- |
| *Lymantria dispar dispar* | LMHRG001-06 | SPI B-5-2 A | HM775749 | Pacific Forestry Centre, Canadian Forest Service | Canada[2] | British Columbia | Present study | N- | B- |
| *Lymantria dispar dispar* | LMHRG002-06 | SPI C-02-02 A | HM775748 | Pacific Forestry Centre, Canadian Forest Service | Canada[2] | British Columbia | Present study | N- | B- |
| *Lymantria dispar dispar* | LMHRG004-06 | SPI C-03-17 A | HM775747 | Pacific Forestry Centre, Canadian Forest Service | Canada[2] | British Columbia | Present study | N- | B- |
| *Lymantria dispar dispar* | LMHRG006-06 | SPI I-3-17 A | HM775746 | Pacific Forestry Centre, Canadian Forest Service | Canada[2] | British Columbia | Present study | N- | B- |
| *Lymantria dispar dispar* | LMHRG007-06 | SPI L-2-5 A | HM775745 | Pacific Forestry Centre, Canadian Forest Service | Canada[2] | British Columbia | Present study | N- | B- |
| *Lymantria dispar dispar* | LMHRG008-06 | SPI V-23-8 A | HM775732 | Pacific Forestry Centre, Canadian Forest Service | Canada[2] | British Columbia | Present study | N- | B- |
| *Lymantria dispar dispar* | LMHRG009-06 | SPI V-23-8 B | HM775731 | Pacific Forestry Centre, Canadian Forest Service | Canada[2] | British Columbia | Present study | N- | B- |
| *Lymantria dispar dispar* | LMHRG010-06 | SPI V-24-3 A | HM775730 | Pacific Forestry Centre, Canadian Forest Service | Canada[2] | British Columbia | Present study | N- | B- |
| *Lymantria dispar dispar* | LMHRG011-06 | SPI V-25-5 A | HM775729 | Pacific Forestry Centre, Canadian Forest Service | Canada[2] | British Columbia | Present study | N- | B- |
| *Lymantria dispar dispar* | LMHRG012-06 | SPI V-26-5 A | HM775728 | Pacific Forestry Centre, Canadian Forest Service | Canada[2] | British Columbia | Present study | N- | B- |
| *Lymantria dispar dispar* | LMHRG014-06 | SPI V-90-2 B | HM775727 | Pacific Forestry Centre, Canadian Forest Service | Canada[2] | British Columbia | Present study | N- | B- |
| *Lymantria dispar dispar* | LMHRG015-06 | SPI V-90-2 A | HM775726 | Pacific Forestry Centre, Canadian Forest Service | Canada[2] | British Columbia | Present study | N- | B- |
| *Lymantria dispar dispar* | LMHRG016-06 | SPI V-90-3 A | HM775725 | Pacific Forestry Centre, Canadian Forest Service | Canada[2] | British Columbia | Present study | N- | B- |
| *Lymantria dispar dispar* | LMHRG019-06 | SPI V-92-1 B | HM775724 | Pacific Forestry Centre, Canadian Forest Service | Canada[2] | British Columbia | Present study | N- | B- |
| *Lymantria dispar dispar* | LMHRG020-06 | SPI V-92-1 C | HM775723 | Pacific Forestry Centre, Canadian Forest Service | Canada[2] | British Columbia | Present study | N- | B- |
| *Lymantria dispar dispar* | LMHRG021-06 | SPI V-92-1 D | HM775722 | Pacific Forestry Centre, Canadian Forest Service | Canada[2] | British Columbia | Present study | N- | B- |
| *Lymantria dispar dispar* | LMHRG022-06 | SPI V-92-1 E | HM775721 | Pacific Forestry Centre, Canadian Forest Service | Canada[2] | British Columbia | Present study | N- | B- |
| *Lymantria dispar dispar* | LMHRG023-06 | SPI V-92-3 A | HM775720 | Pacific Forestry Centre, Canadian Forest Service | Canada[2] | British Columbia | Present study | N- | B- |
| *Lymantria dispar dispar* | LMHRG024-06 | SPI V-92-3 B | HM775719 | Pacific Forestry Centre, Canadian Forest Service | Canada[2] | British Columbia | Present study | N- | B- |
| *Lymantria dispar dispar* | LMHRG025-06 | SPI V-93-1 A | HM775718 | Pacific Forestry Centre, Canadian Forest Service | Canada[2] | British Columbia | Present study | N- | B- |
| *Lymantria dispar dispar* | LMHRG026-06 | SPI V-93-2 A | HM775717 | Pacific Forestry Centre, Canadian Forest Service | Canada[2] | British Columbia | Present study | N- | B- |
| *Lymantria dispar dispar* | LMHRG027-06 | SPI V-93-2 B | HM775716 | Pacific Forestry Centre, Canadian Forest Service | Canada[2] | British Columbia | Present study | N- | B- |

| | | | | | | | | | | |
|---|---|---|---|---|---|---|---|---|---|---|
| *Lymantria dispar dispar* | LMHRG029-06 | SPI V-94-1 B | HM775715 | Pacific Forestry Centre, Canadian Forest Service | Canada[2] | British Columbia | Present study | N- | B- |
| *Lymantria dispar dispar* | LMHRG030-06 | SPI V-94-1 C | HM775714 | Pacific Forestry Centre, Canadian Forest Service | Canada[2] | British Columbia | Present study | N- | B- |
| *Lymantria dispar dispar* | LMHRG031-06 | SPI V-94-1 D | HM775713 | Pacific Forestry Centre, Canadian Forest Service | Canada[2] | British Columbia | Present study | N- | B- |
| *Lymantria dispar dispar* | LMHRG032-06 | SPI V-98-2 A | HM775712 | Pacific Forestry Centre, Canadian Forest Service | Canada[2] | British Columbia | Present study | N- | B- |
| *Lymantria dispar dispar* | LMHRG033-06 | SPI W-17-1 A | HM775711 | Pacific Forestry Centre, Canadian Forest Service | Canada[2] | British Columbia | Present study | N- | B- |
| *Lymantria dispar dispar* | LMHRG035-06 | SPI X-39-1 A | HM775710 | Pacific Forestry Centre, Canadian Forest Service | Canada[2] | British Columbia | Present study | N- | B- |
| *Lymantria dispar dispar* | LMHRG036-06 | KAM DAB-9 A | HM775709 | Pacific Forestry Centre, Canadian Forest Service | Canada[2] | British Columbia | Present study | N- | B- |
| *Lymantria dispar dispar* | LYMAN015-08 | ww01213 | HM775736 | Smithsonian National Museum of Natural History | United States | Virginia | Present study | N- | N/A |
| *Lymantria dispar dispar* | LYMAN016-08 | ww01214 | HM775735 | Smithsonian National Museum of Natural History | United States | Maryland | Present study | N- | B- |
| *Lymantria dispar dispar* | LYMAN017-08 | ww01215 | HM775734 | Smithsonian National Museum of Natural History | United States | Maryland | Present study | N- | N/A |
| *Lymantria dispar dispar* | LYMAN018-08 | ww01216 | HM775733 | Smithsonian National Museum of Natural History | United States | Maryland | Present study | N- | B- |
| *Lymantria dispar dispar* | LYMMK001-09 | LymMk_Q-1 | HM775587 | USDA Forest Service, Northern Research Station | Austria | Vienna | Present study | N+ | B- |
| *Lymantria dispar dispar* | LYMMK002-09 | LymMk_Q-2 | HM775576 | USDA Forest Service, Northern Research Station | Austria | Vienna | Present study | N+ | B- |
| *Lymantria dispar dispar* | LYMMK003-09 | LymMk_Q-3 | HM775565 | USDA Forest Service, Northern Research Station | Austria | Vienna | Present study | N+ | B- |
| *Lymantria dispar dispar* | LYMMK004-09 | LymMk_R-1 | HM775554 | USDA Forest Service, Northern Research Station | Austria | Vienna | Present study | N+ | B- |
| *Lymantria dispar dispar* | LYMMK005-09 | LymMk_R-2 | HM775543 | USDA Forest Service, Northern Research Station | Austria | Vienna | Present study | N+ | B- |
| *Lymantria dispar dispar* | LYMMK006-09 | LymMk_R-3 | HM775620 | USDA Forest Service, Northern Research Station | Austria | Vienna | Present study | N+ | B- |
| *Lymantria dispar dispar* | LYMMK007-09 | LymMk_S-1 | HM775619 | USDA Forest Service, Northern Research Station | Austria | Burgenland | Present study | N+ | B- |
| *Lymantria dispar dispar* | LYMMK008-09 | LymMk_S-2 | HM775699 | USDA Forest Service, Northern Research Station | Austria | Burgenland | Present study | N+ | B- |
| *Lymantria dispar dispar* | LYMMK009-09 | LymMk_S-3 | HM775698 | USDA Forest Service, Northern Research Station | Austria | Burgenland | Present study | N+ | B- |
| *Lymantria dispar dispar* | LYMMK010-09 | LymMk_UB-1 | HM775697 | USDA Forest Service, Northern Research Station | Bulgaria | Sofiya-Grad | Present study | N+ | B- |
| *Lymantria dispar dispar* | LYMMK011-09 | LymMk_UB-2 | HM775612 | USDA Forest Service, Northern Research Station | Bulgaria | Sofiya-Grad | Present study | N+ | B- |
| *Lymantria dispar dispar* | LYMMK012-09 | LymMk_UB-3 | HM775696 | USDA Forest Service, Northern Research Station | Bulgaria | Sofiya-Grad | Present study | N+ | B- |
| *Lymantria dispar dispar* | LYMMK028-09 | LymMk_CCL-1 | HM775683 | USDA Forest Service, Northern Research Station | Croatia | Primorsko-Goranska Zupanjia | Present study | N+ | B- |
| *Lymantria dispar dispar* | LYMMK029-09 | LymMk_CCL-2 | HM775682 | USDA Forest Service, Northern Research Station | Croatia | Primorsko-Goranska Zupanjia | Present study | N+ | B- |
| *Lymantria dispar dispar* | LYMMK030-09 | LymMk_CCL-3 | HM775681 | USDA Forest Service, Northern Research Station | Croatia | Primorsko-Goranska Zupanjia | Present study | N+ | B- |
| *Lymantria dispar dispar* | LYMMK031-09 | LymMk_CSL-1 | HM775680 | USDA Forest Service, Northern Research Station | Slovenia | Sumarija Llrovljani | Present study | N+ | B- |
| *Lymantria dispar dispar* | LYMMK032-09 | LymMk_CSL-2 | HM775679 | USDA Forest Service, Northern Research Station | Slovenia | Sumarija Llrovljani | Present study | N+ | B- |
| *Lymantria dispar dispar* | LYMMK033-09 | LymMk_CSL-3 | HM775678 | USDA Forest Service, Northern Research Station | Slovenia | Sumarija Llrovljani | Present study | N+ | B- |
| *Lymantria dispar dispar* | LYMMK034-09 | LymMk_CSN-1 | HM775677 | USDA Forest Service, Northern Research Station | Slovenia | Sumarija Novska | Present study | N+ | B- |
| *Lymantria dispar dispar* | LYMMK035-09 | LymMk_CSN-2 | HM775676 | USDA Forest Service, Northern Research Station | Slovenia | Sumarija Novska | Present study | N+ | B- |
| *Lymantria dispar dispar* | LYMMK036-09 | LymMk_CSN-3 | HM775675 | USDA Forest Service, Northern Research Station | Slovenia | Sumarija Novska | Present study | N+ | B- |
| *Lymantria dispar dispar* | LYMMK037-09 | LymMk_CSO-1 | HM775674 | USDA Forest Service, Northern Research Station | Slovenia | Sumarija otok | Present study | N+ | B- |
| *Lymantria dispar dispar* | LYMMK038-09 | LymMk_CSO-2 | HM775673 | USDA Forest Service, Northern Research Station | Slovenia | Sumarija otok | Present study | N+ | B- |
| *Lymantria dispar dispar* | LYMMK039-09 | LymMk_CSO-3 | HM775672 | USDA Forest Service, Northern Research Station | Slovenia | Sumarija otok | Present study | N+ | B- |
| *Lymantria dispar dispar* | LYMMK040-09 | LymMk_CSS-1 | HM775671 | USDA Forest Service, Northern Research Station | Slovenia | Sumarija strizovojha | Present study | N+ | B- |
| *Lymantria dispar dispar* | LYMMK041-09 | LymMk_CSS-2 | HM775670 | USDA Forest Service, Northern Research Station | Slovenia | Sumarija strizovojha | Present study | N+ | B- |
| *Lymantria dispar dispar* | LYMMK042-09 | LymMk_CSS-3 | HM775669 | USDA Forest Service, Northern Research Station | Slovenia | Sumarija strizovojha | Present study | N+ | B- |
| *Lymantria dispar dispar* | LYMMK043-09 | LymMk_BF-1 | HM775668 | USDA Forest Service, Northern Research Station | France | Poitou-Charentes | Present study | N+ | B- |
| *Lymantria dispar dispar* | LYMMK044-09 | LymMk_BF-2 | HM775667 | USDA Forest Service, Northern Research Station | France | Poitou-Charentes | Present study | N+ | B- |
| *Lymantria dispar dispar* | LYMMK045-09 | LymMk_BF-3 | HM775666 | USDA Forest Service, Northern Research Station | France | Poitou-Charentes | Present study | N+ | B- |
| *Lymantria dispar dispar* | LYMMK046-09 | LymMk_N-1 | HM775665 | USDA Forest Service, Northern Research Station | France | Indre-et-Loire | Present study | N+ | B- |
| *Lymantria dispar dispar* | LYMMK048-09 | LymMk_O-1 | HM775664 | USDA Forest Service, Northern Research Station | France | Indre-et-Loire | Present study | N+ | B- |
| *Lymantria dispar dispar* | LYMMK049-09 | LymMk_O-2 | HM775663 | USDA Forest Service, Northern Research Station | France | Indre-et-Loire | Present study | N- | B- |
| *Lymantria dispar dispar* | LYMMK050-09 | LymMk_O-3 | HM775662 | USDA Forest Service, Northern Research Station | France | Indre-et-Loire | Present study | N+ | B- |
| *Lymantria dispar dispar* | LYMMK051-09 | LymMk_P-1 | HM775661 | USDA Forest Service, Northern Research Station | France | Indre-et-Loire | Present study | N+ | B- |
| *Lymantria dispar dispar* | LYMMK052-09 | LymMk_P-2 | HM775660 | USDA Forest Service, Northern Research Station | France | Indre-et-Loire | Present study | N+ | B- |
| *Lymantria dispar dispar* | LYMMK053-09 | LymMk_P-3 | HM775659 | USDA Forest Service, Northern Research Station | France | Indre-et-Loire | Present study | N+ | B- |
| *Lymantria dispar dispar* | LYMMK054-09 | LymMk_T-1 | HM775658 | USDA Forest Service, Northern Research Station | France | Alsace | Present study | N- | B- |
| *Lymantria dispar dispar* | LYMMK055-09 | LymMk_T-2 | HM775657 | USDA Forest Service, Northern Research Station | France | Alsace | Present study | N+ | B- |
| *Lymantria dispar dispar* | LYMMK056-09 | LymMk_T-3 | HM775656 | USDA Forest Service, Northern Research Station | France | Alsace | Present study | N+ | B- |
| *Lymantria dispar dispar* | LYMMK057-09 | LymMk_A-1 | HM775655 | USDA Forest Service, Northern Research Station | Germany | Baden-Wuerttemberg | Present study | N+ | B- |
| *Lymantria dispar dispar* | LYMMK058-09 | LymMk_A-2 | HM775654 | USDA Forest Service, Northern Research Station | Germany | Baden-Wuerttemberg | Present study | N+ | B- |
| *Lymantria dispar dispar* | LYMMK059-09 | LymMk_A-3 | HM775653 | USDA Forest Service, Northern Research Station | Germany | Baden-Wuerttemberg | Present study | N+ | B- |
| *Lymantria dispar dispar* | LYMMK060-09 | LymMk_B-1 | HM775652 | USDA Forest Service, Northern Research Station | Germany | Baden-Wuerttemberg | Present study | N+ | B- |
| *Lymantria dispar dispar* | LYMMK061-09 | LymMk_B-2 | HM775651 | USDA Forest Service, Northern Research Station | Germany | Baden-Wuerttemberg | Present study | N+ | B- |
| *Lymantria dispar dispar* | LYMMK062-09 | LymMk_B-3 | HM775650 | USDA Forest Service, Northern Research Station | Germany | Baden-Wuerttemberg | Present study | N+ | B- |
| *Lymantria dispar dispar* | LYMMK063-09 | LymMk_C-1 | HM775649 | USDA Forest Service, Northern Research Station | Germany | Baden-Wuerttemberg | Present study | N+ | B- |
| *Lymantria dispar dispar* | LYMMK064-09 | LymMk_C-2 | HM775648 | USDA Forest Service, Northern Research Station | Germany | Baden-Wuerttemberg | Present study | N+ | B- |
| *Lymantria dispar dispar* | LYMMK065-09 | LymMk_C-3 | HM775647 | USDA Forest Service, Northern Research Station | Germany | Baden-Wuerttemberg | Present study | N+ | B- |
| *Lymantria dispar dispar* | LYMMK066-09 | LymMk_D-1 | HM775646 | USDA Forest Service, Northern Research Station | Germany | Baden-Wuerttemberg | Present study | N+ | B- |
| *Lymantria dispar dispar* | LYMMK067-09 | LymMk_E-1 | HM775645 | USDA Forest Service, Northern Research Station | Germany | Baden-Wuerttemberg | Present study | N+ | B- |
| *Lymantria dispar dispar* | LYMMK068-09 | LymMk_E-2 | HM775644 | USDA Forest Service, Northern Research Station | Germany | Baden-Wuerttemberg | Present study | N+ | B- |
| *Lymantria dispar dispar* | LYMMK069-09 | LymMk_E-3 | HM775643 | USDA Forest Service, Northern Research Station | Germany | Baden-Wuerttemberg | Present study | N+ | B- |
| *Lymantria dispar dispar* | LYMMK070-09 | LymMk_F-1 | HM775642 | USDA Forest Service, Northern Research Station | Germany | Baden-Wuerttemberg | Present study | N+ | B- |
| *Lymantria dispar dispar* | LYMMK071-09 | LymMk_F-2 | HM775641 | USDA Forest Service, Northern Research Station | Germany | Baden-Wuerttemberg | Present study | N+ | B- |
| *Lymantria dispar dispar* | LYMMK072-09 | LymMk_F-3 | HM775640 | USDA Forest Service, Northern Research Station | Germany | Baden-Wuerttemberg | Present study | N+ | B- |
| *Lymantria dispar dispar* | LYMMK073-09 | LymMk_G-1 | HM775606 | USDA Forest Service, Northern Research Station | Germany | Bavaria | Present study | N+ | B- |
| *Lymantria dispar dispar* | LYMMK074-09 | LymMk_G-2 | HM775639 | USDA Forest Service, Northern Research Station | Germany | Bavaria | Present study | N+ | B- |
| *Lymantria dispar dispar* | LYMMK075-09 | LymMk_G-3 | HM775638 | USDA Forest Service, Northern Research Station | Germany | Bavaria | Present study | N+ | B- |
| *Lymantria dispar dispar* | LYMMK076-09 | LymMk_GL-1 | HM775637 | USDA Forest Service, Northern Research Station | Germany | Hesse | Present study | N+ | B- |
| *Lymantria dispar dispar* | LYMMK078-09 | LymMk_GL-3 | HM775636 | USDA Forest Service, Northern Research Station | Germany | Hesse | Present study | N+ | B- |
| *Lymantria dispar dispar* | LYMMK079-09 | LymMk_H-1 | HM775635 | USDA Forest Service, Northern Research Station | Germany | Bavaria | Present study | N+ | B- |
| *Lymantria dispar dispar* | LYMMK080-09 | LymMk_H-2 | HM775634 | USDA Forest Service, Northern Research Station | Germany | Bavaria | Present study | N+ | B- |
| *Lymantria dispar dispar* | LYMMK081-09 | LymMk_H-3 | HM775633 | USDA Forest Service, Northern Research Station | Germany | Bavaria | Present study | N+ | B- |
| *Lymantria dispar dispar* | LYMMK082-09 | LymMk_I-1 | HM775632 | USDA Forest Service, Northern Research Station | Germany | Hesse | Present study | N+ | B- |
| *Lymantria dispar dispar* | LYMMK083-09 | LymMk_I-2 | HM775631 | USDA Forest Service, Northern Research Station | Germany | Hesse | Present study | N+ | B- |
| *Lymantria dispar dispar* | LYMMK084-09 | LymMk_I-3 | HM775630 | USDA Forest Service, Northern Research Station | Germany | Hesse | Present study | N+ | B- |
| *Lymantria dispar dispar* | LYMMK085-09 | LymMk_J-1 | HM775629 | USDA Forest Service, Northern Research Station | Germany | Hesse | Present study | N+ | B- |
| *Lymantria dispar dispar* | LYMMK086-09 | LymMk_J-2 | HM775628 | USDA Forest Service, Northern Research Station | Germany | Hesse | Present study | N+ | B- |
| *Lymantria dispar dispar* | LYMMK087-09 | LymMk_J-3 | HM775627 | USDA Forest Service, Northern Research Station | Germany | Hesse | Present study | N+ | B- |
| *Lymantria dispar dispar* | LYMMK088-09 | LymMk_KG-1 | HM775613 | USDA Forest Service, Northern Research Station | Greece | Curr | Present study | N+ | B- |

| | | | | | | | | | | |
|---|---|---|---|---|---|---|---|---|---|---|
| *Lymantria dispar dispar* | LYMMK089-09 | LymMk_KG-2 | HM775626 | USDA Forest Service, Northern Research Station | Greece | Curr | Present study | N+ | B- |
| *Lymantria dispar dispar* | LYMMK097-09 | LymMk_JL-1 | HM775603 | USDA Forest Service, Northern Research Station | Lithuania | Kuzsin Nezijos | Present study | N+ | B- |
| *Lymantria dispar dispar* | LYMMK098-09 | LymMk_JL-2 | HM775602 | USDA Forest Service, Northern Research Station | Lithuania | Kuzsin Nezijos | Present study | N+ | B- |
| *Lymantria dispar dispar* | LYMMK099-09 | LymMk_JL-3 | HM775601 | USDA Forest Service, Northern Research Station | Lithuania | Kuzsin Nezijos | Present study | N+ | B- |
| *Lymantria dispar dispar* | LYMMK100-09 | LymMk_PA-1 | HM775600 | USDA Forest Service, Northern Research Station | Poland | Poznan Region | Present study | N+ | B- |
| *Lymantria dispar dispar* | LYMMK102-09 | LymMk_PA-3 | HM775599 | USDA Forest Service, Northern Research Station | Poland | Poznan Region | Present study | N+ | B- |
| *Lymantria dispar dispar* | LYMMK103-09 | LymMk_PB-1 | HM775598 | USDA Forest Service, Northern Research Station | Poland | Poznan Region | Present study | N+ | B- |
| *Lymantria dispar dispar* | LYMMK104-09 | LymMk_PB-2 | HM775597 | USDA Forest Service, Northern Research Station | Poland | Poznan Region | Present study | N+ | B- |
| *Lymantria dispar dispar* | LYMMK105-09 | LymMk_PB-3 | HM775596 | USDA Forest Service, Northern Research Station | Poland | Poznan Region | Present study | N+ | B- |
| *Lymantria dispar dispar* | LYMMK106-09 | LymMk_PC-1 | HM775610 | USDA Forest Service, Northern Research Station | Poland | Poznan Region | Present study | N+ | N/A |
| *Lymantria dispar dispar* | LYMMK107-09 | LymMk_PC-2 | HM775609 | USDA Forest Service, Northern Research Station | Poland | Poznan Region | Present study | N+ | N/A |
| *Lymantria dispar dispar* | LYMMK108-09 | LymMk_PC-3 | HM775595 | USDA Forest Service, Northern Research Station | Poland | Poznan Region | Present study | N+ | B- |
| *Lymantria dispar dispar* | LYMMK109-09 | LymMk_PP-1 | HM775594 | USDA Forest Service, Northern Research Station | Portugal | Portalegre | Present study | N+ | B- |
| *Lymantria dispar dispar* | LYMMK110-09 | LymMk_PP-2 | HM775593 | USDA Forest Service, Northern Research Station | Portugal | Portalegre | Present study | N+ | B- |
| *Lymantria dispar dispar* | LYMMK111-09 | LymMk_PP-3 | HM775592 | USDA Forest Service, Northern Research Station | Portugal | Portalegre | Present study | N+ | B- |
| *Lymantria dispar dispar* | LYMMK121-09 | LymMk_ESL-1 | HM775581 | USDA Forest Service, Northern Research Station | Slovakia | Bratislava | Present study | N+ | B- |
| *Lymantria dispar dispar* | LYMMK122-09 | LymMk_ESL-2 | HM775580 | USDA Forest Service, Northern Research Station | Slovakia | Bratislava | Present study | N+ | B- |
| *Lymantria dispar dispar* | LYMMK123-09 | LymMk_ESL-3 | HM775579 | USDA Forest Service, Northern Research Station | Slovakia | Bratislava | Present study | N+ | B- |
| *Lymantria dispar dispar* | LYMMK124-09 | LymMk_K-1 | HM775578 | USDA Forest Service, Northern Research Station | Switzerland | Ticino | Present study | N+ | B- |
| *Lymantria dispar dispar* | LYMMK125-09 | LymMk_K-2 | HM775577 | USDA Forest Service, Northern Research Station | Switzerland | Ticino | Present study | N+ | B- |
| *Lymantria dispar dispar* | LYMMK126-09 | LymMk_K-3 | HM775575 | USDA Forest Service, Northern Research Station | Switzerland | Ticino | Present study | N+ | B- |
| *Lymantria dispar dispar* | LYMMK127-09 | LymMk_L-1 | HM775574 | USDA Forest Service, Northern Research Station | Switzerland | Ticino | Present study | N+ | B- |
| *Lymantria dispar dispar* | LYMMK128-09 | LymMk_L-2 | HM775573 | USDA Forest Service, Northern Research Station | Switzerland | Ticino | Present study | N+ | B- |
| *Lymantria dispar dispar* | LYMMK129-09 | LymMk_L-3 | HM775572 | USDA Forest Service, Northern Research Station | Switzerland | Ticino | Present study | N+ | B- |
| *Lymantria dispar dispar* | LYMMK130-09 | LymMk_M-1 | HM775571 | USDA Forest Service, Northern Research Station | Switzerland | Ticino | Present study | N+ | B- |
| *Lymantria dispar dispar* | LYMMK131-09 | LymMk_M-2 | HM775570 | USDA Forest Service, Northern Research Station | Switzerland | Ticino | Present study | N+ | B- |
| *Lymantria dispar dispar* | LYMMK132-09 | LymMk_M-3 | HM775569 | USDA Forest Service, Northern Research Station | Switzerland | Ticino | Present study | N+ | B- |
| *Lymantria dispar dispar* | LYMMK133-09 | LymMk_NC-1 | HM775568 | USDA Forest Service, Northern Research Station | United States | North Carolina | Present study | N- | B- |
| *Lymantria dispar dispar* | LYMMK134-09 | LymMk_NC-2 | HM775567 | USDA Forest Service, Northern Research Station | United States | North Carolina | Present study | N- | B- |
| *Lymantria dispar dispar* | LYMMK135-09 | LymMk_NC-3 | HM775566 | USDA Forest Service, Northern Research Station | United States | North Carolina | Present study | N- | B- |
| *Lymantria dispar dispar* | LYMMK136-09 | LymMk_CT-1 | HM775564 | USDA Forest Service, Northern Research Station | United States | Connecticut | Present study | N- | B- |
| *Lymantria dispar dispar* | LYMMK137-09 | LymMk_CT-2 | HM775563 | USDA Forest Service, Northern Research Station | United States | Connecticut | Present study | N- | B- |
| *Lymantria dispar dispar* | LYMMK138-09 | LymMk_CT-3 | HM775562 | USDA Forest Service, Northern Research Station | United States | Connecticut | Present study | N- | B- |
| *Lymantria dispar dispar* | LYMMK139-09 | LymMk_CT2-1 | HM775561 | USDA Forest Service, Northern Research Station | United States | Connecticut | Present study | N- | B- |
| *Lymantria dispar dispar* | LYMMK140-09 | LymMk_CT2-2 | HM775560 | USDA Forest Service, Northern Research Station | United States | Connecticut | Present study | N- | B- |
| *Lymantria dispar dispar* | LYMMK141-09 | LymMk_CT2-3 | HM775559 | USDA Forest Service, Northern Research Station | United States | Connecticut | Present study | N- | B- |
| *Lymantria dispar dispar* | LYMMK142-09 | LymMk_LAM-1 | HM775558 | USDA Forest Service, Northern Research Station | United States | New York | Present study | N- | B- |
| *Lymantria dispar dispar* | LYMMK143-09 | LymMk_LAM-2 | HM775557 | USDA Forest Service, Northern Research Station | United States | New York | Present study | N- | B- |
| *Lymantria dispar dispar* | LYMMK144-09 | LymMk_LAM-3 | HM775556 | USDA Forest Service, Northern Research Station | United States | New York | Present study | N- | B- |
| *Lymantria dispar dispar* | LYMMK145-09 | LymMk_LAP-1 | HM775555 | USDA Forest Service, Northern Research Station | United States | New York | Present study | N- | B- |
| *Lymantria dispar dispar* | LYMMK146-09 | LymMk_LAP-2 | HM775553 | USDA Forest Service, Northern Research Station | United States | New York | Present study | N- | B- |
| *Lymantria dispar dispar* | LYMMK147-09 | LymMk_LAP-3 | HM775552 | USDA Forest Service, Northern Research Station | United States | New York | Present study | N- | B- |
| *Lymantria dispar dispar* | LYMMK148-09 | LymMk_LB-1 | HM775551 | USDA Forest Service, Northern Research Station | United States | New York | Present study | N- | B- |
| *Lymantria dispar dispar* | LYMMK149-09 | LymMk_LB-2 | HM775550 | USDA Forest Service, Northern Research Station | United States | New York | Present study | N- | B- |
| *Lymantria dispar dispar* | LYMMK150-09 | LymMk_LB-3 | HM775549 | USDA Forest Service, Northern Research Station | United States | New York | Present study | N- | B- |
| *Lymantria dispar dispar* | LYMMK151-09 | LymMk_LM-1 | HM775548 | USDA Forest Service, Northern Research Station | United States | New York | Present study | N- | B- |
| *Lymantria dispar dispar* | LYMMK152-09 | LymMk_LM-2 | HM775547 | USDA Forest Service, Northern Research Station | United States | New York | Present study | N- | B- |
| *Lymantria dispar dispar* | LYMMK153-09 | LymMk_LM-3 | HM775546 | USDA Forest Service, Northern Research Station | United States | New York | Present study | N- | B- |
| *Lymantria dispar dispar* | LYMMK154-09 | LymMk_LO-1 | HM775545 | USDA Forest Service, Northern Research Station | United States | New York | Present study | N- | B- |
| *Lymantria dispar dispar* | LYMMK155-09 | LymMk_LO-2 | HM775544 | USDA Forest Service, Northern Research Station | United States | New York | Present study | N- | B- |
| *Lymantria dispar dispar* | LYMMK156-09 | LymMk_LO-3 | HM775542 | USDA Forest Service, Northern Research Station | United States | New York | Present study | N- | B- |
| *Lymantria dispar dispar* | LYMMK157-09 | LymMk_MA-1 | HM775541 | USDA Forest Service, Northern Research Station | United States | Massachusetts | Present study | N- | B- |
| *Lymantria dispar dispar* | LYMMK158-09 | LymMk_MA-2 | HM775540 | USDA Forest Service, Northern Research Station | United States | Massachusetts | Present study | N- | B- |
| *Lymantria dispar dispar* | LYMMK159-09 | LymMk_MA-3 | HM775539 | USDA Forest Service, Northern Research Station | United States | Massachusetts | Present study | N- | B- |
| *Lymantria dispar dispar* | LYMMK160-09 | LymMk_WV-1 | HM775538 | USDA Forest Service, Northern Research Station | United States | West Virginia | Present study | N- | B- |
| *Lymantria dispar dispar* | LYMMK161-09 | LymMk_WV-2 | HM775622 | USDA Forest Service, Northern Research Station | United States | West Virginia | Present study | N- | B- |
| *Lymantria dispar dispar* | LYMMK162-09 | LymMk_WV-3 | HM775624 | USDA Forest Service, Northern Research Station | United States | West Virginia | Present study | N- | B- |
| *Lymantria dispar dispar* | LYMMK164-09 | LymMk_LG-2 | HM775621 | USDA Forest Service, Northern Research Station | Latvia | | Present study | N+ | B- |
| *Lymantria dispar dispar* | TMNBD445-07 | MNBTT-3246 | HM775744 | Biodiversity Institute of Ontario | Canada | New Brunswick | Present study | N- | B- |
| *Lymantria dispar dispar* | TMNBD446-07 | MNBTT-3247 | HM775743 | Biodiversity Institute of Ontario | Canada | New Brunswick | Present study | N- | B- |
| *Lymantria dispar dispar* | TMNBD447-07 | MNBTT-3248 | HM775742 | Biodiversity Institute of Ontario | Canada | New Brunswick | Present study | N- | B- |
| *Lymantria dispar dispar* | TMNBD448-07 | MNBTT-3249 | HM775741 | Biodiversity Institute of Ontario | Canada | New Brunswick | Present study | N- | B- |
| *Lymantria dispar dispar* | TTMNB259-06 | MNBTT-259 | HM775740 | Biodiversity Institute of Ontario | Canada | New Brunswick | Present study | N- | B- |
| *Lymantria dispar dispar* | TTMNB260-06 | MNBTT-260 | HM775739 | Biodiversity Institute of Ontario | Canada | New Brunswick | Present study | N- | B- |
| *Lymantria dispar dispar* | XAG005-05 | 2005-ONT-589 | GU091217 | Biodiversity Institute of Ontario | Canada | Ontario | Hebert et al. 2010 | N- | B- |
| *Lymantria dispar dispar* | XAG177-05 | 2005-ONT-761 | GU091216 | Biodiversity Institute of Ontario | Canada | Ontario | Hebert et al. 2010 | N- | B- |
| *Lymantria dispar dispar* | XAG214-05 | 2005-ONT-798 | GU091222 | Biodiversity Institute of Ontario | Canada | Ontario | Hebert et al. 2010 | N- | B- |
| *Lymantria dispar dispar* | XAG215-05 | 2005-ONT-799 | GU091219 | Biodiversity Institute of Ontario | Canada | Ontario | Hebert et al. 2010 | N- | B- |
| *Lymantria dispar dispar* | XAG216-05 | 2005-ONT-800 | GU091220 | Biodiversity Institute of Ontario | Canada | Ontario | Hebert et al. 2010 | N- | B- |
| *Lymantria dispar dispar* | XAG217-05 | 2005-ONT-801 | GU091221 | Biodiversity Institute of Ontario | Canada | Ontario | Hebert et al. 2010 | N- | B- |
| *Lymantria dispar dispar* | XAG235-05 | 2005-ONT-819 | GU091223 | Biodiversity Institute of Ontario | Canada | Ontario | Hebert et al. 2010 | N- | B- |
| *Lymantria dispar dispar* | XAG277-05 | 2005-ONT-861 | GU091215 | Biodiversity Institute of Ontario | Canada | Ontario | Hebert et al. 2010 | N- | B- |
| *Lymantria dispar dispar* | XAG307-05 | 2005-ONT-891 | GU091213 | Biodiversity Institute of Ontario | Canada | Ontario | Hebert et al. 2010 | N- | B- |
| *Lymantria dispar dispar* | XAG308-05 | 2005-ONT-892 | GU091214 | Biodiversity Institute of Ontario | Canada | Ontario | Hebert et al. 2010 | N- | B- |
| *Lymantria dispar dispar* | XAG348-05 | 2005-ONT-932 | GU091218 | Biodiversity Institute of Ontario | Canada | Ontario | Hebert et al. 2010 | N- | B- |
| *Lymantria dispar dispar* | XAG658-05 | 2005-ONT-1242 | GU091224 | Biodiversity Institute of Ontario | Canada | Ontario | Hebert et al. 2010 | N- | B- |
| *Lymantria dispar dispar* | XAK267-06 | 2006-ONT-1262 | HM775738 | Biodiversity Institute of Ontario | Canada | Ontario | Present study | N- | B- |
| *Lymantria dispar dispar* | XAK268-06 | 2006-ONT-1263 | HM775737 | Biodiversity Institute of Ontario | Canada | Ontario | Present study | N- | B- |
| *Lymantria dispar japonica* | BOGDA038-08 | Bogda-JA1-38 | N/A[1] | Unknown (GenBank, NCBI) | Japan | Kyushu-chiho | Bogdanowicz et al. 2000 | N+ | B+ |
| *Lymantria dispar japonica* | BOGDA039-08 | Bogda-JA1-39 | N/A[1] | Unknown (GenBank, NCBI) | Japan | Kyushu-chiho | Bogdanowicz et al. 2000 | N+ | B+ |
| *Lymantria dispar japonica* | BOGDA040-08 | Bogda-JA2-40 | N/A[1] | Unknown (GenBank, NCBI) | Japan | Kyushu-chiho | Bogdanowicz et al. 2000 | N+ | B+ |
| *Lymantria dispar japonica* | BOGDA041-08 | Bogda-JA2-41 | N/A[1] | Unknown (GenBank, NCBI) | Japan | Kyushu-chiho | Bogdanowicz et al. 2000 | N+ | B+ |

| | | | | | | | | | | |
|---|---|---|---|---|---|---|---|---|---|---|
| *Lymantria dispar japonica* | BOGDA042-08 | Bogda-JA3-42 | N/A[1] | Unknown (GenBank, NCBI) | Japan | Honshu | Bogdanowicz et al. 2000 | N+ | B+ |
| *Lymantria dispar japonica* | BOGDA043-08 | Bogda-JA4-43 | N/A[1] | Unknown (GenBank, NCBI) | Japan | Honshu | Bogdanowicz et al. 2000 | N+ | B+ |
| *Lymantria dispar japonica* | BOGDA044-08 | Bogda-JA4-44 | N/A[1] | Unknown (GenBank, NCBI) | Japan | Honshu | Bogdanowicz et al. 2000 | N+ | B+ |
| *Lymantria dispar japonica* | BOGDA052-08 | Bogda-JA6-52 | N/A[1] | Unknown (GenBank, NCBI) | Japan | Honshu | Bogdanowicz et al. 2000 | N+ | B+ |
| *Lymantria dispar japonica* | BOGDA053-08 | Bogda-JA6-53 | N/A[1] | Unknown (GenBank, NCBI) | Japan | Honshu | Bogdanowicz et al. 2000 | N+ | B- |
| *Lymantria dispar japonica* | BOGDA054-08 | Bogda-JA7-54 | N/A[1] | Unknown (GenBank, NCBI) | Japan | Honshu | Bogdanowicz et al. 2000 | N+ | B+ |
| *Lymantria dispar japonica* | BOGDA055-08 | Bogda-JA8-55 | N/A[1] | Unknown (GenBank, NCBI) | Japan | Honshu | Bogdanowicz et al. 2000 | N+ | B+ |
| *Lymantria dispar japonica* | BOGDA056-08 | Bogda-JA8-56 | N/A[1] | Unknown (GenBank, NCBI) | Japan | Honshu | Bogdanowicz et al. 2000 | N+ | B+ |
| *Lymantria dispar japonica* | BOGDA057-08 | Bogda-JA9-57 | N/A[1] | Unknown (GenBank, NCBI) | Japan | Honshu | Bogdanowicz et al. 2000 | N+ | B+ |
| *Lymantria dispar japonica* | BOGDA058-08 | Bogda-JA9-58 | N/A[1] | Unknown (GenBank, NCBI) | Japan | Honshu | Bogdanowicz et al. 2000 | N+ | B+ |
| *Lymantria dispar japonica* | GBGL1527-06 | Ly321 | DQ116110 | Unknown (GenBank, NCBI) | Japan | Honshu | Armstrong & Ball 2005 | N+ | B+ |
| *Lymantria dispar japonica* | GBGL1528-06 | LY323 | DQ116111 | Unknown (GenBank, NCBI) | Japan | Honshu | Armstrong & Ball 2005 | N+ | B+ |
| *Lymantria dispar japonica* | GBGL1542-06 | Ly332 | DQ116125 | Unknown (GenBank, NCBI) | Japan | Honshu | Armstrong & Ball 2005 | N+ | B+ |
| *Lymantria dispar japonica* | GBGL1548-06 | Ly326 | DQ116131 | Unknown (GenBank, NCBI) | Japan | Honshu | Armstrong & Ball 2005 | N+ | B+ |
| *Lymantria dispar japonica* | GBGL1553-06 | Ly328 | DQ116136 | Unknown (GenBank, NCBI) | Japan | Honshu | Armstrong & Ball 2005 | N+ | B+ |
| *Lymantria dispar japonica* | GBGL1570-06 | Ly337 | DQ116153 | Unknown (GenBank, NCBI) | Japan | Honshu | Armstrong & Ball 2005 | N+ | B+ |
| *Lymantria dispar japonica* | LYMAN007-08 | ww01205 | HM775753 | Smithsonian National Museum of Natural History | Japan | Honshu | Present study | N+ | B+ |
| *Lymantria dispar japonica* | LYMAN008-08 | ww01206 | HM775752 | Smithsonian National Museum of Natural History | Japan | Honshu | Present study | N+ | B+ |
| *Lymantria dispar japonica* | LYMAN009-08 | ww01207 | HM775751 | Smithsonian National Museum of Natural History | Japan | Honshu | Present study | N+ | N/A |
| *Lymantria dispar japonica* | LYMAN010-08 | ww01208 | HM775750 | Smithsonian National Museum of Natural History | Japan | Honshu | Present study | N+ | N/A |
| *Lymantria dispar japonica* | LYMMK091-09 | LymMk_Hon-1 | HM775625 | USDA Forest Service, Northern Research Station | Japan | Honshu | Present study | N+ | B+ |
| *Lymantria dispar japonica* | LYMMK092-09 | LymMk_Hon-2 | HM775611 | USDA Forest Service, Northern Research Station | Japan | Honshu | Present study | N+ | B+ |
| *Lymantria dispar japonica* | LYMMK093-09 | LymMk_Hon-3 | HM775604 | USDA Forest Service, Northern Research Station | Japan | Honshu | Present study | N+ | B+ |
| *Lymantria dissoluta* | LYMAN055-08 | ww01253 | HM775755 | Smithsonian National Museum of Natural History | China | Guangdong | Present study | N+ | N/A |
| *Lymantria dissoluta* | LYMAN071-08 | ww01269 | HM775754 | Smithsonian National Museum of Natural History | Hong Kong | N.T. Shatin | Present study | N+ | N/A |
| *Lymantria dissoluta* | LYMAN181-08 | ww02259 | HM775756 | Wagga Wagga Agricultural Institute | China | Fujian | Present study | N+ | N/A |
| *Lymantria ekeikei* | LYMAN096-08 | ww01294 | HM775757 | Smithsonian National Museum of Natural History | Indonesia | Irian Jaya | Present study | N+ | N/A |
| *Lymantria flavida* | GBGL1504-06 | Ly144a | DQ116087 | Unknown (GenBank, NCBI) | Japan | Okinawa | Armstrong & Ball 2005 | N+ | B- |
| *Lymantria flavida* | GBGL1505-06 | Ly166 | DQ116088 | Unknown (GenBank, NCBI) | Japan | Okinawa | Armstrong & Ball 2005 | N+ | B- |
| *Lymantria flavida* | LYMAN026-08 | ww01224 | HM775761 | Smithsonian National Museum of Natural History | Japan | Okinawa | Present study | N+ | B- |
| *Lymantria flavida* | LYMAN027-08 | ww01225 | HM775760 | Smithsonian National Museum of Natural History | Japan | Okinawa | Present study | N+ | B- |
| *Lymantria flavida* | LYMAN028-08 | ww01226 | HM775759 | Smithsonian National Museum of Natural History | Japan | Okinawa | Present study | N+ | B- |
| *Lymantria flavida* | LYMAN029-08 | ww01227 | HM775758 | Smithsonian National Museum of Natural History | Japan | Okinawa | Present study | N+ | B- |
| *Lymantria fumida* | GBGL1589-06 | Ly248 | DQ116172 | Unknown (GenBank, NCBI) | Japan | Honshu | Armstrong & Ball 2005 | N+ | B- |
| *Lymantria fumida* | GBGL1607-06 | Lepi679 | DQ116190 | Unknown (GenBank, NCBI) | Japan | Honshu | Armstrong & Ball 2005 | N+ | B- |
| *Lymantria fumida* | GBGL1608-06 | Ly248_2 | DQ116191 | Unknown (GenBank, NCBI) | Japan | Honshu | Armstrong & Ball 2005 | N+ | B- |
| *Lymantria fumida* | LYMAN075-08 | ww01273 | HM775764 | Smithsonian National Museum of Natural History | Japan | Iwate | Present study | N+ | B- |
| *Lymantria fumida* | LYMAN085-08 | ww01283 | HM775763 | Smithsonian National Museum of Natural History | Japan | Honshu | Present study | N+ | B- |
| *Lymantria fumida* | LYMAN086-08 | ww01284 | HM775762 | Smithsonian National Museum of Natural History | Japan | Honshu | Present study | N+ | N/A |
| *Lymantria grisea* | LYMAN097-08 | ww01295 | HM775765 | Smithsonian National Museum of Natural History | India | Assam | Present study | N+ | B- |
| *Lymantria lucescens* | GBGL1594-06 | Ly184 | DQ116177 | Unknown (GenBank, NCBI) | Japan | Honshu | Armstrong & Ball 2005 | N- | B- |
| *Lymantria lucescens* | GBGL1613-06 | Lep1114 | DQ149569 | Unknown (GenBank, NCBI) | Japan | Honshu | Ball & Armstrong 2006 | N- | B- |
| *Lymantria lucescens* | LYMAN063-08 | ww01261 | HM775767 | Smithsonian National Museum of Natural History | Japan | Honshu | Present study | N- | N/A |
| *Lymantria lucescens* | LYMAN064-08 | ww01262 | HM775766 | Smithsonian National Museum of Natural History | Japan | Honshu | Present study | N- | N/A |
| *Lymantria mathura* | GBGL1574-06 | Ly99 | DQ116157 | Unknown (GenBank, NCBI) | South Korea | Kangwon-do | Armstrong & Ball 2005 | N- | B- |
| *Lymantria mathura* | GBGL1576-06 | Ly121 | DQ116159 | Unknown (GenBank, NCBI) | South Korea | Kangwon-do | Armstrong & Ball 2005 | N- | B- |
| *Lymantria mathura* | GBGL1577-06 | Ly123 | DQ116160 | Unknown (GenBank, NCBI) | South Korea | Kangwon-do | Armstrong & Ball 2005 | N- | B- |
| *Lymantria mathura* | GBGL1578-06 | Ly125 | DQ116161 | Unknown (GenBank, NCBI) | South Korea | Yongau-ri | Armstrong & Ball 2005 | N- | B- |
| *Lymantria mathura* | LYMAN019-08 | ww01217 | HM775789 | Smithsonian National Museum of Natural History | Japan | Hokkaido | Present study | N- | B- |
| *Lymantria mathura* | LYMAN020-08 | ww01218 | HM775788 | Smithsonian National Museum of Natural History | Japan | Honshu | Present study | N- | N/A |
| *Lymantria mathura* | LYMAN021-08 | ww01219 | HM775787 | Smithsonian National Museum of Natural History | Japan | Honshu | Present study | N- | N/A |
| *Lymantria mathura* | LYMAN022-08 | ww01220 | HM775786 | Smithsonian National Museum of Natural History | South Korea | nr. Soraksan NP Campground | Present study | N- | B- |
| *Lymantria mathura* | LYMAN030-08 | ww01228 | HM775785 | Smithsonian National Museum of Natural History | Japan | Hokkaido | Present study | N- | B- |
| *Lymantria mathura* | LYRFE002-08 | PaA-08-1182 | HM775774 | Pacific Forestry Centre, Canadian Forest Service | Japan | Fukushima | Present study | N- | B- |
| *Lymantria mathura* | LYRFE003-08 | PaA-08-1183 | HM775773 | Pacific Forestry Centre, Canadian Forest Service | Japan | Fukushima | Present study | N- | B- |
| *Lymantria mathura* | LYRFE004-08 | PaA-08-1184 | HM775772 | Pacific Forestry Centre, Canadian Forest Service | Japan | Fukushima | Present study | N- | B- |
| *Lymantria mathura* | LYRFE005-08 | PaA-08-1185 | HM775771 | Pacific Forestry Centre, Canadian Forest Service | Japan | Fukushima | Present study | N- | B- |
| *Lymantria mathura* | LYRFE006-08 | PaA-08-1186 | HM775770 | Pacific Forestry Centre, Canadian Forest Service | Japan | Fukushima | Present study | N- | B- |
| *Lymantria mathura* | LYRFE007-08 | PaA-08-1187 | HM775769 | Pacific Forestry Centre, Canadian Forest Service | Japan | Fukushima | Present study | N- | B- |
| *Lymantria mathura* | LYRFE008-08 | PaA-08-1188 | HM775768 | Pacific Forestry Centre, Canadian Forest Service | Japan | Fukushima | Present study | N- | B- |
| *Lymantria mathura* | RFELP001-08 | PaA-08-524 | HM775784 | Pacific Forestry Centre, Canadian Forest Service | Russia | Primorskiy Kray | Present study | N- | N/A |
| *Lymantria mathura* | RFELP002-08 | PaA-08-525 | HM775783 | Pacific Forestry Centre, Canadian Forest Service | Russia | Primorskiy Kray | Present study | N- | B- |
| *Lymantria mathura* | RFELP003-08 | PaA-08-526 | HM775782 | Pacific Forestry Centre, Canadian Forest Service | Russia | Primorskiy Kray | Present study | N- | B- |
| *Lymantria mathura* | RFELP004-08 | PaA-08-527 | HM775781 | Pacific Forestry Centre, Canadian Forest Service | Russia | Primorskiy Kray | Present study | N- | B- |
| *Lymantria mathura* | RFELP005-08 | PaA-08-528 | HM775780 | Pacific Forestry Centre, Canadian Forest Service | Russia | Primorskiy Kray | Present study | N- | B- |
| *Lymantria mathura* | RFELP006-08 | PaA-08-529 | HM775779 | Pacific Forestry Centre, Canadian Forest Service | Russia | Primorskiy Kray | Present study | N- | B- |
| *Lymantria mathura* | RFELP007-08 | PaA-08-530 | HM775778 | Pacific Forestry Centre, Canadian Forest Service | Russia | Primorskiy Kray | Present study | N- | B- |
| *Lymantria mathura* | RFELP008-08 | PaA-08-531 | HM775777 | Pacific Forestry Centre, Canadian Forest Service | Russia | Primorskiy Kray | Present study | N- | B- |
| *Lymantria mathura* | RFELP009-08 | PaA-08-532 | HM775776 | Pacific Forestry Centre, Canadian Forest Service | Russia | Primorskiy Kray | Present study | N- | B- |
| *Lymantria mathura* | RFELP010-08 | PaA-08-533 | HM775775 | Pacific Forestry Centre, Canadian Forest Service | Russia | Primorskiy Kray | Present study | N- | B- |
| *Lymantria minomonis* | LTOLB230-09 | AYK-04-5397 | HM775790 | University of Maryland | Japan | Yamanashi-ken | Present study | N+ | B- |
| *Lymantria monacha* | BOGDA071-08 | Bogda-MON-71 | N/A[1] | Unknown (GenBank, NCBI) | Japan | Hokkaido | Bogdanowicz et al. 2000 | N+ | B- |
| *Lymantria monacha* | GBGL1506-06 | Ly247 | DQ116089 | Unknown (GenBank, NCBI) | Japan | Honshu | Armstrong & Ball 2005 | N+ | B- |
| *Lymantria monacha* | GBGL1508-06 | Ly44_2 | DQ116091 | Unknown (GenBank, NCBI) | Czech Republic | | Armstrong & Ball 2005 | N+ | B- |
| *Lymantria monacha* | GBGL1512-06 | Ly65_2 | DQ116095 | Unknown (GenBank, NCBI) | Poland | | Armstrong & Ball 2005 | N+ | B- |
| *Lymantria monacha* | GBGL1514-06 | Ly389 | DQ116097 | Unknown (GenBank, NCBI) | Japan | Honshu | Armstrong & Ball 2005 | N+ | B- |
| *Lymantria monacha* | GBGL1518-06 | Ly385 | DQ116101 | Unknown (GenBank, NCBI) | Japan | Honshu | Armstrong & Ball 2005 | N+ | B- |
| *Lymantria monacha* | GBGL1519-06 | Ly392 | DQ116102 | Unknown (GenBank, NCBI) | Japan | Honshu | Armstrong & Ball 2005 | N+ | B- |
| *Lymantria monacha* | GBGL1530-06 | Ly394 | DQ116113 | Unknown (GenBank, NCBI) | Japan | Honshu | Armstrong & Ball 2005 | N+ | B- |
| *Lymantria monacha* | GBGL1531-06 | Ly395 | DQ116114 | Unknown (GenBank, NCBI) | Japan | Honshu | Armstrong & Ball 2005 | N+ | B- |
| *Lymantria monacha* | GBGL1540-06 | Ly69 | DQ116123 | Unknown (GenBank, NCBI) | Czech Republic | | Armstrong & Ball 2005 | N+ | B- |
| *Lymantria monacha* | GBGL1541-06 | Ly239 | DQ116124 | Unknown (GenBank, NCBI) | Japan | Honshu | Armstrong & Ball 2005 | N+ | B- |

| | | | | | | | | | |
|---|---|---|---|---|---|---|---|---|---|
| *Lymantria monacha* | GBGL1544-06 | Ly43 | DQ116127 | Unknown (GenBank, NCBI) | Poland | | Armstrong & Ball 2005 | N+ | B- |
| *Lymantria monacha* | GBGL1546-06 | Ly67 | DQ116129 | Unknown (GenBank, NCBI) | Czech Republic | | Armstrong & Ball 2005 | N+ | B- |
| *Lymantria monacha* | GBGL1547-06 | Ly70 | DQ116130 | Unknown (GenBank, NCBI) | Czech Republic | | Armstrong & Ball 2005 | N+ | B- |
| *Lymantria monacha* | GBGL1549-06 | Ly396 | DQ116132 | Unknown (GenBank, NCBI) | Japan | Honshu | Armstrong & Ball 2005 | N+ | B- |
| *Lymantria monacha* | GBGL1551-06 | Ly68 | DQ116134 | Unknown (GenBank, NCBI) | Czech Republic | | Armstrong & Ball 2005 | N+ | B- |
| *Lymantria monacha* | GBGL1552-06 | Ly238 | DQ116135 | Unknown (GenBank, NCBI) | Japan | Honshu | Armstrong & Ball 2005 | N+ | B- |
| *Lymantria monacha* | GBGL1554-06 | Ly401 | DQ116137 | Unknown (GenBank, NCBI) | Japan | Honshu | Armstrong & Ball 2005 | N+ | B- |
| *Lymantria monacha* | GBGL1560-06 | Ly64 | DQ116143 | Unknown (GenBank, NCBI) | Poland | | Armstrong & Ball 2005 | N+ | B- |
| *Lymantria monacha* | GBGL1561-06 | Ly65 | DQ116144 | Unknown (GenBank, NCBI) | Poland | | Armstrong & Ball 2005 | N+ | B- |
| *Lymantria monacha* | GBGL1562-06 | Ly202 | DQ116145 | Unknown (GenBank, NCBI) | South Korea | Kangwon-do | Armstrong & Ball 2005 | N+ | B- |
| *Lymantria monacha* | GBGL1563-06 | Ly203 | DQ116146 | Unknown (GenBank, NCBI) | South Korea | Kangwon-do | Armstrong & Ball 2005 | N+ | B- |
| *Lymantria monacha* | GBGL1564-06 | Ly208 | DQ116147 | Unknown (GenBank, NCBI) | South Korea | Kangwon-do | Armstrong & Ball 2005 | N+ | B- |
| *Lymantria monacha* | GBGL1565-06 | Ly214 | DQ116148 | Unknown (GenBank, NCBI) | South Korea | Kangwon-do | Armstrong & Ball 2005 | N+ | B- |
| *Lymantria monacha* | GBGL1566-06 | Ly217 | DQ116149 | Unknown (GenBank, NCBI) | South Korea | Kangwon-do | Armstrong & Ball 2005 | N+ | B- |
| *Lymantria monacha* | GBGL1571-06 | Ly342 | DQ116154 | Unknown (GenBank, NCBI) | Japan | Honshu | Armstrong & Ball 2005 | N+ | B- |
| *Lymantria monacha* | GBGL1572-06 | Ly347 | DQ116155 | Unknown (GenBank, NCBI) | Japan | Honshu | Armstrong & Ball 2005 | N+ | B- |
| *Lymantria monacha* | GBGL1573-06 | Ly343 | DQ116156 | Unknown (GenBank, NCBI) | Japan | Honshu | Armstrong & Ball 2005 | N+ | B- |
| *Lymantria monacha* | GBGL1581-06 | Ly240 | DQ116164 | Unknown (GenBank, NCBI) | Japan | Honshu | Armstrong & Ball 2005 | N+ | B- |
| *Lymantria monacha* | GBGL4527-07 | AF075277 | AF075277 | Unknown (GenBank, NCBI) | Japan | Hokkaido | Bogdanowicz et al. 2000 | N+ | B- |
| *Lymantria monacha* | LYMMK172-09 | LymMk_CZP-1 | HM775818 | USDA Forest Service, Northern Research Station | Czech Republic | | Present study | N+ | B- |
| *Lymantria monacha* | LYMMK173-09 | LymMk_CZP-2 | HM775817 | USDA Forest Service, Northern Research Station | Czech Republic | | Present study | N+ | B- |
| *Lymantria monacha* | LYMMK174-09 | LymMk_CZP-3 | HM775816 | USDA Forest Service, Northern Research Station | Czech Republic | | Present study | N+ | B- |
| *Lymantria monacha* | LYMMK175-09 | LymMk_CZP-4 | HM775791 | USDA Forest Service, Northern Research Station | Czech Republic | | Present study | N+ | N/A |
| *Lymantria monacha* | LYMMK176-09 | LymMk_CZP-5 | HM775815 | USDA Forest Service, Northern Research Station | Czech Republic | | Present study | N+ | B- |
| *Lymantria monacha* | LYMMK177-09 | LymMk_CZP-6 | HM775814 | USDA Forest Service, Northern Research Station | Czech Republic | | Present study | N+ | B- |
| *Lymantria monacha* | LYMMK178-09 | LymMk_JIP-1 | HM775813 | USDA Forest Service, Northern Research Station | Japan | | Present study | N+ | B- |
| *Lymantria monacha* | LYMMK179-09 | LymMk_JIP-2 | HM775812 | USDA Forest Service, Northern Research Station | Japan | | Present study | N+ | B- |
| *Lymantria monacha* | LYMMK180-09 | LymMk_JIP-3 | HM775811 | USDA Forest Service, Northern Research Station | Japan | | Present study | N+ | B- |
| *Lymantria monacha* | LYMMK181-09 | LymMk_JIP-4 | HM775810 | USDA Forest Service, Northern Research Station | Japan | | Present study | N+ | B- |
| *Lymantria monacha* | RFELP011-08 | PaA-08-534 | HM775807 | Pacific Forestry Centre, Canadian Forest Service | Russia | Primorskiy Kray | Present study | N+ | B- |
| *Lymantria monacha* | RFELP012-08 | PaA-08-535 | HM775806 | Pacific Forestry Centre, Canadian Forest Service | Russia | Primorskiy Kray | Present study | N+ | N/A |
| *Lymantria monacha* | RFELP013-08 | PaA-08-536 | HM775805 | Pacific Forestry Centre, Canadian Forest Service | Russia | Primorskiy Kray | Present study | N+ | B- |
| *Lymantria monacha* | RFELP014-08 | PaA-08-537 | HM775808 | Pacific Forestry Centre, Canadian Forest Service | Russia | Primorskiy Kray | Present study | N+ | B- |
| *Lymantria monacha* | RFELP015-08 | PaA-08-538 | HM775804 | Pacific Forestry Centre, Canadian Forest Service | Russia | Primorskiy Kray | Present study | N+ | B- |
| *Lymantria monacha* | RFELP016-08 | PaA-08-539 | HM775803 | Pacific Forestry Centre, Canadian Forest Service | Russia | Primorskiy Kray | Present study | N+ | B- |
| *Lymantria monacha* | RFELP017-08 | PaA-08-540 | HM775802 | Pacific Forestry Centre, Canadian Forest Service | Russia | Primorskiy Kray | Present study | N+ | B- |
| *Lymantria monacha* | RFELP018-08 | PaA-08-541 | HM775801 | Pacific Forestry Centre, Canadian Forest Service | Russia | Primorskiy Kray | Present study | N+ | B- |
| *Lymantria monacha* | RFELP019-08 | PaA-08-542 | HM775800 | Pacific Forestry Centre, Canadian Forest Service | Russia | Primorskiy Kray | Present study | N+ | N/A |
| *Lymantria monacha* | RFELP020-08 | PaA-08-543 | HM775799 | Pacific Forestry Centre, Canadian Forest Service | Russia | Primorskiy Kray | Present study | N+ | B- |
| *Lymantria monacha* | RFELP021-08 | PaA-08-544 | HM775798 | Pacific Forestry Centre, Canadian Forest Service | Russia | Primorskiy Kray | Present study | N+ | B- |
| *Lymantria monacha* | RFELP022-08 | PaA-08-545 | HM775809 | Pacific Forestry Centre, Canadian Forest Service | Russia | Primorskiy Kray | Present study | N+ | B- |
| *Lymantria monacha* | RFELP023-08 | PaA-08-546 | HM775797 | Pacific Forestry Centre, Canadian Forest Service | Russia | Primorskiy Kray | Present study | N+ | B- |
| *Lymantria monacha* | RFELP024-08 | PaA-08-547 | HM775796 | Pacific Forestry Centre, Canadian Forest Service | Russia | Primorskiy Kray | Present study | N+ | B- |
| *Lymantria monacha* | RFELP025-08 | PaA-08-548 | HM775795 | Pacific Forestry Centre, Canadian Forest Service | Russia | Primorskiy Kray | Present study | N+ | N/A |
| *Lymantria monacha* | RFELP026-08 | PaA-08-549 | HM775794 | Pacific Forestry Centre, Canadian Forest Service | Russia | Primorskiy Kray | Present study | N+ | N/A |
| *Lymantria monacha* | RFELP027-08 | PaA-08-550 | HM775793 | Pacific Forestry Centre, Canadian Forest Service | Russia | Primorskiy Kray | Present study | N+ | B- |
| *Lymantria monacha* | RFELP028-08 | PaA-08-551 | HM775792 | Pacific Forestry Centre, Canadian Forest Service | Russia | Primorskiy Kray | Present study | N+ | N/A |
| *Lymantria naesigi* | LYMAN101-08 | ww01299 | HM775819 | Smithsonian National Museum of Natural History | Philippines | Negros Occidental | Present study | N+ | B- |
| *Lymantria narindra* | LYMAN093-08 | ww01291 | HM775820 | Smithsonian National Museum of Natural History | Thailand | Near Thongphaphem | Present study | N- | B- |
| *Lymantria nebulosa* | LYMAN104-08 | ww01302 | HM775821 | Smithsonian National Museum of Natural History | China | Wuzhi Shan | Present study | N+ | B+ |
| *Lymantria nephrographa* | LYMAN094-08 | ww01292 | HM775822 | Smithsonian National Museum of Natural History | Australia | New South Wales | Present study | N- | N/A |
| *Lymantria ninayi* | HCHL034-04 | USNM ENT 196233 | HM775823 | Smithsonian National Museum of Natural History | Papua New Guinea | Eastern Highlands | Present study | N+ | B- |
| *Lymantria ninayi* | HCHL035-04 | USNM ENT 196232 | HM775824 | Smithsonian National Museum of Natural History | Papua New Guinea | Eastern Highlands | Present study | N+ | B- |
| *Lymantria obfuscata* | BOGDA067-08 | Bogda-OBF-67 | N/A[1] | Unknown (GenBank, NCBI) | India | Kashmir | Bogdanowicz et al. 2000 | N+ | B- |
| *Lymantria obfuscata* | GBGL1603-06 | Lepi484 | DQ116186 | Unknown (GenBank, NCBI) | India | | Armstrong & Ball 2005 | N+ | B- |
| *Lymantria obfuscata* | GBGL1604-06 | Lepi485 | DQ116187 | Unknown (GenBank, NCBI) | India | | Armstrong & Ball 2005 | N- | B- |
| *Lymantria obfuscata* | GBGL1605-06 | Lepi486 | DQ116188 | Unknown (GenBank, NCBI) | India | | Armstrong & Ball 2005 | N+ | B- |
| *Lymantria obfuscata* | GBGL4529-07 | OBF | AF075275 | Unknown (GenBank, NCBI) | India | Jammu and Kashmir | Bogdanowicz et al. 2000 | N+ | B- |
| *Lymantria obfuscata* | GBGL4970-08 | Lepi1024 | DQ155599 | Unknown (GenBank, NCBI) | India | Himachal Pradesh | Ball & Armstrong 2006 | N+ | B- |
| *Lymantria obfuscata* | LYMAN073-08 | ww01271 | HM775826 | Smithsonian National Museum of Natural History | India | Kulu Val | Present study | N+ | B- |
| *Lymantria obfuscata* | LYMAN074-08 | ww01272 | HM775825 | Smithsonian National Museum of Natural History | India | Kulu Val | Present study | N+ | B- |
| *Lymantria panthera* | LYMAN099-08 | ww01297 | HM775827 | Smithsonian National Museum of Natural History | Indonesia | Kalimantan Selatan | Present study | N+ | B- |
| *Lymantria plumbalis* | LYMAN106-08 | ww01304 | HM775836 | Smithsonian National Museum of Natural History | Thailand | Pakchong | Present study | N- | N/A |
| *Lymantria plumbalis* | LYMAN107-08 | ww01305 | HM775835 | Smithsonian National Museum of Natural History | Thailand | Pakchong | Present study | N- | B- |
| *Lymantria plumbalis* | LYMAN108-08 | ww01306 | HM775834 | Smithsonian National Museum of Natural History | Thailand | Pakchong | Present study | N- | B- |
| *Lymantria plumbalis* | LYMAN109-08 | ww01307 | HM775833 | Smithsonian National Museum of Natural History | Thailand | Pakchong | Present study | N- | B- |
| *Lymantria plumbalis* | LYMAN110-08 | ww01308 | HM775832 | Smithsonian National Museum of Natural History | Thailand | Pakchong | Present study | N- | B- |
| *Lymantria plumbalis* | LYMAN111-08 | ww01309 | HM775831 | Smithsonian National Museum of Natural History | Thailand | Pakchong | Present study | N- | B- |
| *Lymantria plumbalis* | LYMAN112-08 | ww01310 | HM775830 | Smithsonian National Museum of Natural History | Thailand | Pakchong | Present study | N- | B- |
| *Lymantria plumbalis* | LYMAN113-08 | ww01311 | HM775829 | Smithsonian National Museum of Natural History | Thailand | Pakchong | Present study | N- | B- |
| *Lymantria plumbalis* | LYMAN114-08 | ww01312 | HM775828 | Smithsonian National Museum of Natural History | Thailand | Pakchong | Present study | N- | B- |
| *Lymantria pulverea* | LYMAN082-08 | ww01280 | HM775838 | Smithsonian National Museum of Natural History | Taiwan | Tayuling | Present study | N+ | N/A |
| *Lymantria pulverea* | LYMAN083-08 | ww01281 | HM775837 | Smithsonian National Museum of Natural History | Taiwan | Tayuling | Present study | N+ | B- |
| *Lymantria rhapdota* | LYMAN105-08 | ww01303 | HM775839 | Smithsonian National Museum of Natural History | Philippines | N. Palawan | Present study | N+ | N/A |
| *Lymantria schaeferi* | LYMAN187-08 | ww02265 | HM775840 | Wagga Wagga Agricultural Institute | China | Fujian | Present study | N+ | B- |
| *Lymantria semperi* | LYMAN185-08 | ww02263 | HM775841 | Wagga Wagga Agricultural Institute | Philippines | Mindoro | Present study | N/A | N/A |
| *Lymantria singapura* | LYMAN179-08 | ww02257 | HM775842 | Wagga Wagga Agricultural Institute | China | Yunnan | Present study | N/A | N/A |
| *Lymantria sinica* | LYMAN065-08 | ww01263 | HM775845 | Smithsonian National Museum of Natural History | Taiwan | T`ao-yuan Chen | Present study | N+ | N/A |
| *Lymantria sinica* | LYMAN066-08 | ww01264 | HM775844 | Smithsonian National Museum of Natural History | Taiwan | T`ao-yuan Chen | Present study | N+ | B- |
| *Lymantria sinica* | LYMAN070-08 | ww01268 | HM775843 | Smithsonian National Museum of Natural History | China | Hongkong | Present study | N+ | B- |
| *Lymantria subpallida* | LYMAN023-08 | ww01221 | HM775848 | Smithsonian National Museum of Natural History | Taiwan | Wulai | Present study | N+ | N/A |
| *Lymantria subpallida* | LYMAN024-08 | ww01222 | HM775847 | Smithsonian National Museum of Natural History | Taiwan | Wulai | Present study | N+ | N/A |

| | | | | | | | | | |
|---|---|---|---|---|---|---|---|---|---|
| *Lymantria* subpallida | LYMAN025-08 | ww01223 | HM775846 | Smithsonian National Museum of Natural History | Hong Kong | Shek Kong | Present study | N+ | N/A |
| *Lymantria subrosea* | LYMAN068-08 | ww01266 | HM775849 | Smithsonian National Museum of Natural History | Sri Lanka | Mate. Dist. | Present study | N/A | N/A |
| *Lymantria todara* | LYMAN189-08 | ww02267 | HM775850 | Wagga Wagga Agricultural Institute | India | Trivendrum | Present study | N/A | N/A |
| *Lymantria umbrifera* | LYMAN059-08 | ww01257 | HM775852 | Smithsonian National Museum of Natural History | Taiwan | Pilushi | Present study | N- | N/A |
| *Lymantria umbrifera* | LYMAN060-08 | ww01258 | HM775851 | Smithsonian National Museum of Natural History | Taiwan | Pilushi | Present study | N- | N/A |
| *Lymantria umbrosa* | BOGDA059-08 | Bogda-JA10-59 | N/A[1] | Unknown (GenBank, NCBI) | Japan | Hokkaido | Bogdanowicz et al. 2000 | N+ | B- |
| *Lymantria umbrosa* | BOGDA060-08 | Bogda-JA10-60 | N/A[1] | Unknown (GenBank, NCBI) | Japan | Hokkaido | Bogdanowicz et al. 2000 | N+ | B- |
| *Lymantria umbrosa* | BOGDA061-08 | Bogda-JA10-61 | N/A[1] | Unknown (GenBank, NCBI) | Japan | Hokkaido | Bogdanowicz et al. 2000 | N+ | B- |
| *Lymantria umbrosa* | BOGDA062-08 | Bogda-JA10-62 | N/A[1] | Unknown (GenBank, NCBI) | Japan | Hokkaido | Bogdanowicz et al. 2000 | N+ | B- |
| *Lymantria umbrosa* | BOGDA063-08 | Bogda-JA10-63 | N/A[1] | Unknown (GenBank, NCBI) | Japan | Hokkaido | Bogdanowicz et al. 2000 | N+ | B- |
| *Lymantria umbrosa* | GBGL1543-06 | Ly419 | DQ116126 | Unknown (GenBank, NCBI) | Japan | Hokkaido | Armstrong & Ball 2005 | N+ | B- |
| *Lymantria umbrosa* | GBGL1584-06 | Ly420 | DQ116167 | Unknown (GenBank, NCBI) | Japan | Hokkaido | Armstrong & Ball 2005 | N+ | B- |
| *Lymantria umbrosa* | GBGL1596-06 | Ly475 | DQ116179 | Unknown (GenBank, NCBI) | Japan | Hokkaido | Armstrong & Ball 2005 | N+ | B- |
| *Lymantria umbrosa* | GBGL1597-06 | Ly477 | DQ116180 | Unknown (GenBank, NCBI) | Japan | Hokkaido | Armstrong & Ball 2005 | N+ | B- |
| *Lymantria umbrosa* | GBGL1598-06 | Ly479 | DQ116181 | Unknown (GenBank, NCBI) | Japan | Hokkaido | Armstrong & Ball 2005 | N+ | B- |
| *Lymantria umbrosa* | GBGL1599-06 | Ly480 | DQ116182 | Unknown (GenBank, NCBI) | Japan | Hokkaido | Armstrong & Ball 2005 | N+ | B- |
| *Lymantria umbrosa* | GBGL4423-07 | AB244668 | AB244668 | Unknown (GenBank, NCBI) | Japan | Hokkaido | Yamaguchi et al. unpublished | N+ | B- |
| *Lymantria umbrosa* | GBGL4424-07 | AB244667 | AB244667 | Unknown (GenBank, NCBI) | Japan | Hokkaido | Yamaguchi et al. unpublished | N+ | B- |
| *Lymantria umbrosa* | GBGL4425-07 | AB244666 | AB244666 | Unknown (GenBank, NCBI) | Japan | Hokkaido | Yamaguchi et al. unpublished | N+ | B- |
| *Lymantria umbrosa* | GBGL4426-07 | AB244664 | AB244664 | Unknown (GenBank, NCBI) | Japan | Hokkaido | Yamaguchi et al. unpublished | N+ | B- |
| *Lymantria umbrosa* | GBGL4427-07 | AB244661 | AB244661 | Unknown (GenBank, NCBI) | Japan | Hokkaido | Yamaguchi et al. unpublished | N+ | B- |
| *Lymantria umbrosa* | GBGL4429-07 | AB244652 | AB244652 | Unknown (GenBank, NCBI) | Japan | Hokkaido | Yamaguchi et al. unpublished | N+ | B- |
| *Lymantria umbrosa* | GBGL4431-07 | AB244657 | AB244657 | Unknown (GenBank, NCBI) | Japan | Hokkaido | Yamaguchi et al. unpublished | N+ | B- |
| *Lymantria umbrosa* | GBGL4531-07 | AF075273 | AF075273 | Unknown (GenBank, NCBI) | Japan | Hokkaido | Bogdanowicz et al. 2000 | N+ | B- |
| *Lymantria umbrosa* | LYMMK095-09 | LymMk_JJ-2 | HM775853 | USDA Forest Service, Northern Research Station | Japan | Hokkaido | Present study | N+ | B- |
| *Lymantria umbrosa* | LYMMK096-09 | LymMk_JJ-3 | HM775854 | USDA Forest Service, Northern Research Station | Japan | Hokkaido | Present study | N+ | B- |
| *Lymantria xylina* | BOGDA069-08 | Bogda-XYL-69 | N/A[1] | Unknown (GenBank, NCBI) | Taiwan | Taipei | Bogdanowicz et al. 2000 | N+ | B- |
| *Lymantria xylina* | GBGL1575-06 | Ly104 | DQ116158 | Unknown (GenBank, NCBI) | Taiwan | Kuan-in | Armstrong & Ball 2005 | N+ | B- |
| *Lymantria xylina* | GBGL1587-06 | Ly105 | DQ116170 | Unknown (GenBank, NCBI) | Taiwan | Kuan-in | Armstrong & Ball 2005 | N+ | B- |
| *Lymantria xylina* | GBGL1588-06 | Ly106 | DQ116171 | Unknown (GenBank, NCBI) | Taiwan | Kuan-in | Armstrong & Ball 2005 | N+ | B- |
| *Lymantria xylina* | GBGL4528-07 | AF075276 | AF075276 | Unknown (GenBank, NCBI) | Taiwan | T`ai-pei Hsien | Bogdanowicz et al. 2000 | N+ | B- |
| | | | | | | | | | |

[1] Only a few representative sequences were deposited in GenBank from Bogdanowicz et al. 2000; the remaining sequences were manually constructed based on their text and figures

[2] This specimen was collected as part of a gypsy moth surveillance program and the country and locality refers to this event
