# Supplementary material for: Towards a Global Barcode Library for Lymantria (Lepidoptera: Lymantriinae) Tussock Moths of Biosecurity Concern
Source: PLoS One. 2010 Dec 9;5(12):e14280. doi: 10.1371/journal.pone.0014280 (PMC3000334; doi:10.1371/journal.pone.0014280)
Supplement: Appendix S1 — Taxonomic treatment of Lymantria nebulosa Wileman, revised status and Lymantria subpallida Okano, revised status. (9.23 MB PDF) [file pone.0014280.s001.pdf]

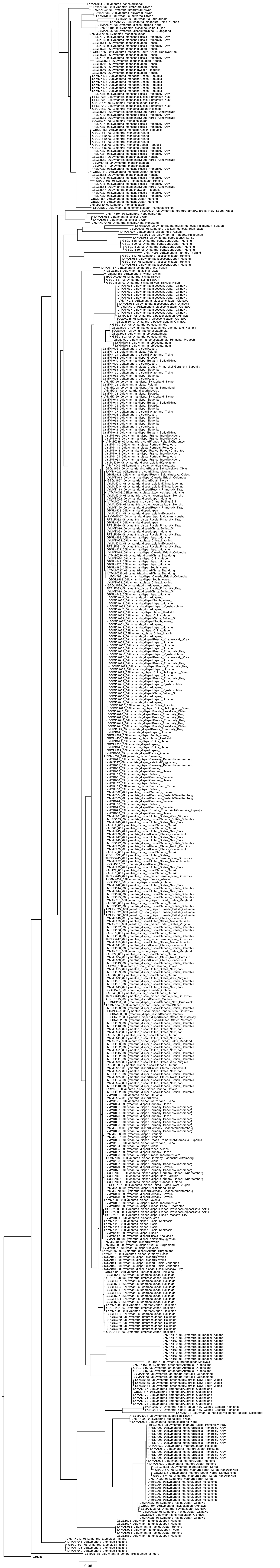
Orgyia
0.05
